# Illuminating the catalytic core of ectoine synthase through structural and biochemical analysis

**DOI:** 10.1038/s41598-018-36247-w

**Published:** 2019-01-23

**Authors:** Laura Czech, Astrid Höppner, Stefanie Kobus, Andreas Seubert, Ramona Riclea, Jeroen S. Dickschat, Johann Heider, Sander H. J. Smits, Erhard Bremer

**Affiliations:** 1 Department of Biology, Laboratory for Microbiology, Philipps-University Marburg, D-35043 Marburg, Germany; 2 Center for Structural Studies, Heinrich-Heine-University Düsseldorf, D-40225 Düsseldorf, Germany; 3 Department of Chemistry, Analytical Chemistry, Philipps-University Marburg, D-35043 Marburg, Germany; 4 Kekulé-Institute for Organic Chemistry and Biochemistry, Friedrich-Wilhelms-University Bonn, D-53121 Bonn, Germany; 5 LOEWE Center for Synthetic Microbiology, Philipps-University Marburg, D-35043 Marburg, Germany; 6 Institute of Biochemistry, Heinrich-Heine-University Düsseldorf, D-40225 Düsseldorf, Germany

## Abstract

Ectoine synthase (EctC) is the signature enzyme for the production of ectoine, a compatible solute and chemical chaperone widely synthesized by bacteria as a cellular defense against the detrimental effects of osmotic stress. EctC catalyzes the last step in ectoine synthesis through cyclo-condensation of the EctA-formed substrate *N*-gamma-acetyl-L-2,4-diaminobutyric acid via a water elimination reaction. We have biochemically and structurally characterized the EctC enzyme from the thermo-tolerant bacterium *Paenibacillus lautus* (*Pl*). EctC is a member of the cupin superfamily and forms dimers, both in solution and in crystals. We obtained high-resolution crystal structures of the (*Pl*)EctC protein in forms that contain (i) the catalytically important iron, (ii) iron and the substrate *N*-gamma-acetyl-L-2,4-diaminobutyric acid, and (iii) iron and the enzyme reaction product ectoine. These crystal structures lay the framework for a proposal for the EctC-mediated water-elimination reaction mechanism. Residues involved in coordinating the metal, the substrate, or the product within the active site of ectoine synthase are highly conserved among a large group of EctC-type proteins. Collectively, the biochemical, mutational, and structural data reported here yielded detailed insight into the structure-function relationship of the (*Pl*)EctC enzyme and are relevant for a deeper understanding of the ectoine synthase family as a whole.

## Introduction

**Figure 1:**
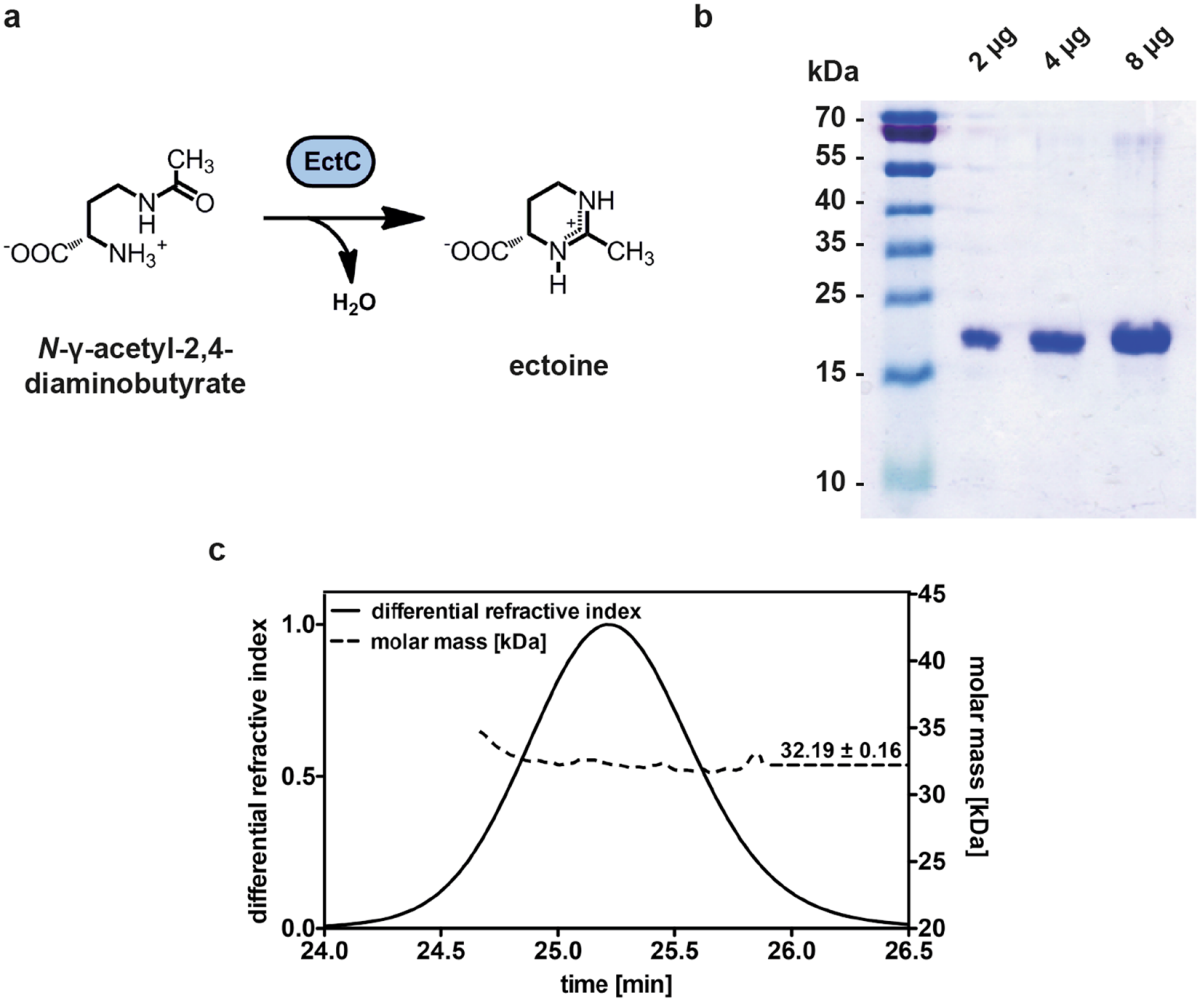
Ectoine synthase-catalyzed enzyme reaction scheme, purification and quaternary assembly of the (*Pl*)EctC protein. (**a**) Scheme of the ectoine synthase catalyzed cyclo-condensation of the substrate *N*-γ-ADABA to form ectoine. (**b**) Coomassie brilliant blue stained 15% SDS-polyacrylamide gel with different amounts of the affinity chromatography purified (*Pl*)EctC-*Strep*-tag II protein. The PageRuler Prestained Protein Ladder (Thermo Scientific) was used as a sizing marker. A picture of the original SDS-gel can be found in Fig. S1. (**c**) SEC-MALS analysis to determine the quaternary assembly of the purified (*Pl*)EctC-*Strep*-tag II protein.

Ectoine [(*S*)−2-methyl-1,4,5,6-tetrahydropyrimidine-4-carboxylic acid]^1^ (Fig. 1a) and its derivative 5-hydroxyectoine [(4*S*, 5*S*)−5-hydroxy-2-methyl-1,4,5,6-tetrahydropyrimidine-4-carboxylic acid]^2^ are representatives of a specialized group of organic osmolytes, the compatible solutes^3–5^. These types of compounds are widely used by members of each domain of life as effective cytoprotectants^6^, in particular against the detrimental effects caused by high external salinity or osmolarity on cellular hydration, physiology, and growth. Compatible solutes are well suited for this demanding task^3,7,8^ because their physico-chemical attributes make them compliant with cellular biochemistry^9–11^. The function-preserving properties of these solutes allow their high-level cellular accumulation, a process raising the osmotic potential of the cytoplasm, which then in turn counteracts the high osmolarity-instigated efflux of water from the cell^3,12^. At the same time, the solvent properties of the crowded cytoplasm are optimized for vital biochemical reactions^13–15^, so that growth can occur under osmotically unfavorable circumstances^3,7,16^. Compatible solutes also serve as stabilizers of proteins, macromolecular assemblies, and even entire cells, both *in vitro* and *in vivo*^11,17–24^, a property that led to their description in the literature as chemical  chaperones^25,26^.

With the notable exception of their recent discovery in a restricted group of obligate halophilic protists^27–29^ and a few *Archaea*^30^, ectoines are primarily synthesized by members of the *Bacteria*^31^, as judged from the wide occurrence of the ectoine (*ectABC*)^32^ and 5-hydroxyectoine (*ectD*)^33^ biosynthetic genes in microbial genome sequences^5^. Biosynthesis of ectoine proceeds from L-aspartate-β-semialdehyde, a central hub in bacterial amino acid and cell wall biosynthesis^34,35^ and is catalyzed by the sequential enzyme reactions of L-2,4-diaminobutyrate transaminase (EctB; EC 2.6.1.76), L-2,4-diaminobutyrate acetyltransferase (EctA; EC 2.3.1.178), and ectoine synthase (EctC; EC 4.2.1.108)^32,36^. 5-Hydroxyectoine is synthesized from ectoine by the ectoine hydroxylase (EctD; EC 1.14.11.55)^33,37,38^ through a regio- and stereo-specific hydroxylation reaction^33^. In comparison with ectoine, this chemical modification endows 5-hydroxyectoine with enhanced, or additional, cellular protection against selected environmental challenges (e.g., heat and desiccation stress)^21,22,39,40^. High-level expression of the ectoine/5-hydroxyectoine biosynthetic genes is typically triggered by increases in the external osmolarity and sometimes also by extremes in either high or low growth temperatures^4,41–43^. Disruption of the *ectABC*(*D*) genes causes sensitivity to such environmental challenges^39,44^, highlighting the prominent role played by ectoines in the stress management of microbial cells^4,5,45^.

The macromolecule stabilizing, cytoprotective, and anti-inflammatory effects of ectoines have generated considerable interest in the commercial and medical use of these natural products and led to an industrial scale biotechnological production process that delivers ectoines on the scale of tons. This process relies on the salt-tolerant bacterium *Halomonas elongata* as a natural production host^4,45–47^. In addition, recombinant cell factories are increasingly developed to offer additional means to produce these valuable compounds biotechnologically^5,48–56^. Hence, both from the perspective of practical applications and basic science^4,5,45^, a deeper understanding of the enzymatic and structural properties of the ectoine and 5-hydroxyectoine biosynthetic enzymes is desirable. This has already been achieved to a considerable degree for the ectoine hydroxylase (EctD)^31,37,38,57^, a member of the non-heme-containing iron(II) and 2-oxoglutarate-dependent dioxygenases enzyme super-family^58^.

In comparison with EctD, the ectoine biosynthetic enzymes (EctABC)^36,59^ are biochemically and structurally far less understood. While the EctA and EctB enzymes have amino acid sequence-related counterparts in various biochemical pathways in microorganisms^5,32,36^, the ectoine synthase (EctC) is considered the diagnostic enzyme for ectoine production^30,31,60^. It can be used in database searches of microbial genome sequences to identify potential ectoine producers^5^. EctC catalyzes the final step in the ectoine biosynthetic route and cyclizes the linear, EctA-produced, *N*-γ-acetyl-L-2,4-diaminobutyric acid (*N*-γ-ADABA) to ectoine through a water elimination reaction^36,60^ (Fig. 1a). Basic biochemical characterizations of ectoine synthases from the extremophiles *H. elongata, Methylomicrobium alcaliphilum*, and *Acidiphilium cryptum*^36,61,62^, the cold-adapted marine bacterium *Sphingopyxis alaskensis*^60^, and the nitrifying archaeon *Nitrosopumilus maritimus*^30^ have been carried out, but a deeper understanding of this enzyme is lacking.

Crystallographic analysis of the psychrophilic *S. alaskensis* EctC protein [(*Sa*)EctC] showed that ectoine synthase is a member of the cupin superfamily^60^. This family comprises a large group of pro- and eukaryotic proteins built on a common structural scaffold; its members can perform a variety of both enzymatic and non-enzymatic functions^63–65^. Most of these proteins contain catalytically important divalent metals (e.g., iron, copper, zinc, manganese, cobalt, or nickel) that allow different types of chemistry to occur within the confines of an evolutionarily conserved tertiary structure^66,67^. Biochemical studies conducted with the (*Sa*)EctC protein revealed for the first time that ectoine synthase is also a metal-dependent enzyme, with Fe^2+^ as the physiologically most likely relevant catalyst^60^. This finding has obvious ramifications with respect to the details of the not yet fully understood reaction mechanism catalyzed by the EctC enzyme (Fig. 1a).

One of our major aims is to understand the structure-function relationship of the ectoine synthase through biochemical and crystallographic analysis. In this respect, the structure of the (*Sa*)EctC protein that we reported recently^60^ provided only restricted functional information because it contained neither the catalytically important metal, nor the substrate or the reaction product. To significantly advance our understanding of the key enzyme for ectoine biosynthesis, we explored the EctC protein from the thermo-tolerant bacterium *Paenibacillus lautus* (*Pl*)^68^ for biochemical and structural studies. We now report here crystallographic views into the catalytic core of the ectoine synthase prior and subsequent to enzyme catalysis. These crystal structures lay the framework for a proposal for the EctC-mediated cyclo-condensation reaction mechanism. Since the residues participating in metal, substrate, and reaction product binding are highly conserved among a very large collection of EctC-type proteins, the data provided here for the (*Pl*)EctC enzyme are relevant for a structural and functional understanding of the extended ectoine synthase family as a whole.

## Results

### Overproduction, purification, and oligomeric state of the (*Pl*)EctC ectoine synthase

Ectoine biosynthetic genes are present in microorganisms able to colonize ecological niches with rather different physico-chemical attributes^4,5,30,31^. One of these ectoine-producing microorganisms is *Paenibacillus lautus* (*Pl*) strain Y4.12MC10, a Gram-positive spore-forming bacterium that was originally isolated from the effluent of the Obsidian hot spring in the Yellowstone Natural Park (USA). It can grow under laboratory conditions up to 50 °C^68^. We therefore explored the EctC protein from *P. lautus* for biochemical and structural studies in the hope that the properties of the ectoine synthase from this thermo-tolerant strain might be more suitable for crystallographic analysis than the psychrophilic EctC protein derived from the cold-adapted bacterium *S. alaskensis* (*Sa*)^69^ which yielded crystal structures only in its apo-form^60^. The (*Pl*)EctC protein possesses 130 amino acids, has a calculated molecular mass of 14.7 kDa, and possesses a calculated isoelectric point of 4.7.

We constructed an expression vector carrying a codon-optimized version of the *P. lautus ectC* gene for its heterologous expression in *Escherichia coli* that would lead to a recombinant protein fused at its C-terminus to a *Strep*-tag II affinity peptide [(*Pl*)EctC*-Strep*-tag II] allowing its purification by affinity chromatography (Figs 1b and S1). The quaternary assembly of the purified (*Pl*)EctC*-Strep*-tag II protein was assessed by size exclusion chromatography followed by multi-angle light scattering (SEC-MALS); these experiments yielded a value for the molecular mass of 32.2 kDa of the (*Pl*)EctC*-Strep*-tag II protein in solution (Fig. 1c). Since the calculated molecular mass for the recombinant (*Pl*)EctC*-Strep*-tag II protein is 15.87 kDa, the ectoine synthase from *P. lautus* is a dimer in solution.

### Biochemical and kinetic properties of the recombinant (*Pl*)EctC enzyme

**Figure 2:**
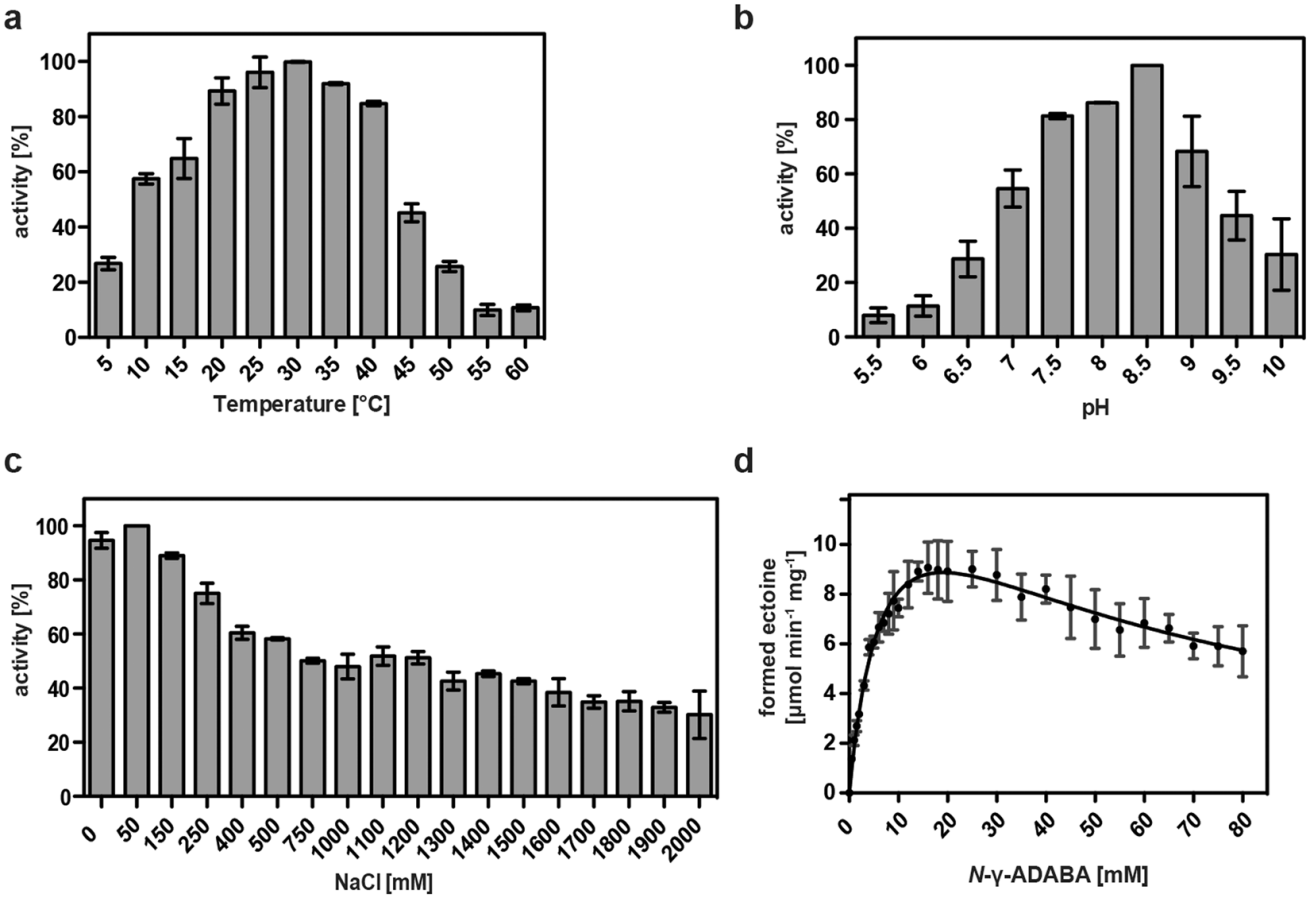
Biochemical properties and kinetic parameters of the *P. lautus* ectoine synthase. The enzyme activity of the affinity-purified (*Pl*)EctC protein was assessed with respect to its temperature (**a**) and pH optima (**b**), and its tolerance against NaCl (**c**). For these enzyme assays 10 mM of the substrate *N*-γ-ADABA and 1 µg of the (*Pl*)EctC protein were used and the assays were run for 5 min at 30 °C. (**d**) Kinetic parameters of the (*Pl*)EctC enzyme were determined in an optimized buffer solution [20 mM HEPES buffer (pH 8.5), 50 mM NaCl, 0.1 mM (NH_4_)_2_Fe(SO_4_)_2_] using 1 µg of the protein and increasing concentrations of the substrate *N*-γ-ADABA. The enzyme assays were conducted at 30 °C and were run for 2.5 min. Formation of ectoine was monitored by HPLC analysis and quantitated as described^30,41,60^. The data shown represent experiments with three independently purified (*Pl*)EctC protein preparations, and each data-point from the individual protein preparations was assayed twice.

Taking into account that the (*Sa*)EctC enzyme is an iron-dependent enzyme^60^, we included 0.1 mM (NH_4_)_2_Fe(SO_4_)_2_ into the buffer solution when we assessed its enzymatic activity. We first determined some basic biochemical properties of the recombinant (*Pl*)EctC enzyme with respect to its temperature and pH optima and its tolerance towards salt (Fig. 2a–c). In keeping with the thermo-tolerant physiology of the *P. lautus* Y4.12MC10 donor strain^68^, the (*Pl*)EctC enzyme exhibited a broad window of temperatures in which it could function. Its temperature optimum was about 30 °C but the purified (*Pl*)EctC enzyme retained 45% and 26% of its activity at 45 °C and 50 °C, respectively, under the tested conditions (Fig. 2a). The (*Pl*)EctC enzyme had an alkaline pH optimum of 8.5 (Fig. 2b) and was highly NaCl tolerant, allowing the protein to retain even 30% of its activity when the assay buffer contained elevated levels (2 M) of NaCl (Fig. 2c).

Building on these initial biochemical assessments of the (*Pl*)EctC protein, we formulated an optimized enzyme activity assay [20 mM HEPES buffer (pH 8.5), 50 mM NaCl, 0.1 mM (NH_4_)_2_Fe(SO_4_)_2_] to determine the kinetic properties of the *P. lautus* ectoine synthase (Fig. 2d). For these assays we used a chemically synthesized and highly purified preparation of *N*-γ-ADABA^60^, the natural substrate of the EctC enzyme^36,59^ (Fig. 1a). The (*Pl*)EctC protein had the following kinetic parameters: (i) a *K*_m_ of 7.8 ± 1.0 mM and (ii) a calculated theoretical *V*_max_ of 16.0 ± 1.2 μmol ectoine formed min^−1^ mg protein^−1^ (Fig. 2d) since the activity of the (*Pl*)EctC enzyme displayed a substantial substrate (or product) inhibition (calculated *K*_i_ of 47 ± 7 mM). The actually measured *V*_max_ of the (*Pl*)EctC enzyme is approximately 9 μmol ectoine formed min^−1^ mg protein^−1^.

Kinetic characterization of EctC enzyme from five other microorganisms have previously been carried out and the following values for ectoine synthase activity were reported: a *K*_m_ of 11 mM and a *V*_max_ of 85 μmol min^−1^ mg^−1^ for the *H. elongata* enzyme^36^ [the parameters for the backward reaction of this enzyme, the hydrolysis of the synthetic ectoine derivative homoectoine, were as follows: a *K*_m_ of 28.7 mM and *V*_max_ of 4.6 μmol min^−1^ mg^−1^]^70^, a *V*_max_ of 64 μmol min^−1^ mg^−1^ for the *M. alcaliphilum* enzyme^71^, a *K*_m_ of 5 mM and a *V*_max_ of 25 μmol min^−1^ mg^−1^ for the *S. alaskensis* enzyme^60^, and a *K*_m_ of 6.4 mM and a *V*_max_ of 12.8 μmol min^−1^ mg^−1^ for the *N. maritimus* enzyme^30^. Preliminary kinetic data were reported for the *A. cryptum* enzyme^61^. Hence, the mentioned ectoine synthases display in some cases notable differences in their kinetic properties but it should be noted that the various EctC enzymes were assessed under rather different buffer, pH, temperature, and salt concentrations (and partially also at different levels of purity). This makes a direct comparison with the kinetic parameters determined in this study for the (*Pl*)EctC enzyme difficult. We also note in this context that the *Pl*)EctC enzyme is the first ectoine synthase for which a substantial substrate (or product) inhibition was detected (Fig. 2d).

### Crystallization of the ectoine synthase in complex with iron, the substrate *N*-γ-ADABA, and the enzyme reaction product ectoine

Crystals of the (*Pl*)EctC protein were obtained using commercial screens and by slightly optimizing the composition of the crystallization solution. Crystals were grown in a solution consisting of 0.2 M ammonium sulfate, 0.1 M phosphate citrate (pH 4.2), 20% (v/v) PEG 300, and 10% (v/v) glycerol. Since the ectoine synthase is an iron dependent enzyme^60^, we added Fe(II)Cl_2_ to the crystallization solution of the purified *(Pl)*EctC protein to a final concentration of 4 mM and pre-incubated this mixture for at least 30 minutes prior to crystallization trials. Without this pre-incubation in the presence of iron, no crystals with a quality suitable for X-ray analysis could be grown. This approach yielded crystals which diffracted to a maximum of 1.6 Å for the *(Pl)*EctC::Fe protein complex (Table 1). To obtain crystals of the (*Pl*)EctC enzyme with its substrate *N*-γ-ADABA or its reaction product ectoine, we added these compounds to a final concentration of 40 mM and of 20 mM, respectively, to *(Pl)*EctC preparations pre-incubated with iron. Crystals which diffracted to a maximum of 2.0 Å were obtained for the *(Pl)*EctC::Fe/*N*-γ-ADABA complex and 2.5 Å for the (*Pl)*EctC::Fe/ectoine complex (Table 1).

**Table 1:** Data collection, phasing and refinement statistics for (*Pl*)EctC::Fe, (*Pl*)EctC::Fe/*N*-γ-ADABA, and (*Pl*)EctC::Fe/ectoine, respectively.

	(*Pl*)EctC::Fe (PDB entry: 5ONM)	(*Pl*)EctC::Fe/*N*-γ-ADABA (PDB entry: 5ONN)	(*Pl*)EctC::Fe/ectoine (PDB entry: 5ONO)
**Data collection**	P13 DESY Hamburg	P13 DESY Hamburg	P13 DESY Hamburg
Wavelength (Å)	0.953	0.953	0.953
Space group	*P 31 2 1*	*P 31 2 1*	*P 31 2 1*
**Cell dimensions**			
a, b, c (Å)	71.13; 71.13; 68.66	70.91; 70.91; 68.54	71.41; 71.41; 68.83
α, β, γ (°)	90.0; 90.0; 120.0	90.0; 90.0; 120.0	90.0; 90.0; 120.0
**Processing**			
Resolution (Å)	35.56–1.6 (1.7–1.6)	31.49–2.0 (2.1–2.0)	46.01–2.5 (2.6–2.5)
*R*_*merge*_	6.5 (45.2)	4.5 (6.89)	8.2 (17.6)
Mean I/sigma (I)	19.14 (1.70)	61.45 (47.39)	8.2 (3.2)
Completeness (%)	99.35 (97.83)	98.05 (97.58)	99.32 (98.33)
Total reflections	586136 (34379)	293346 (29757)	145322 (15326)
Unique reflections	31125 (3015)	13580 (1328)	7282 (706)
Redundancy	18.8 (11.4)	21.6 (22.4)	5.4 (3.0)
**Refinement**			
Resolution (Å)	35.56–1.6	31.49–2.0	46.01–2.5
No. of reflections	545106	281608	36963
R _work_/R _free_	0.17/0.20	0.17/0.21	0.18/0.24
**RMSD**			
Bond length (Å)	0.011	0.016	0.014
Bond angles (°)	1.41	1.80	1.48
**No. atoms**			
Protein	1115	1115	1115
Ligand	6	12	12
Water	133	152	42
***B*** **-factors**			
Protein (Å)	22.80	21.60	20.70
Ligand (Å)	31.20	35.20	86.10

**Figure 3:**
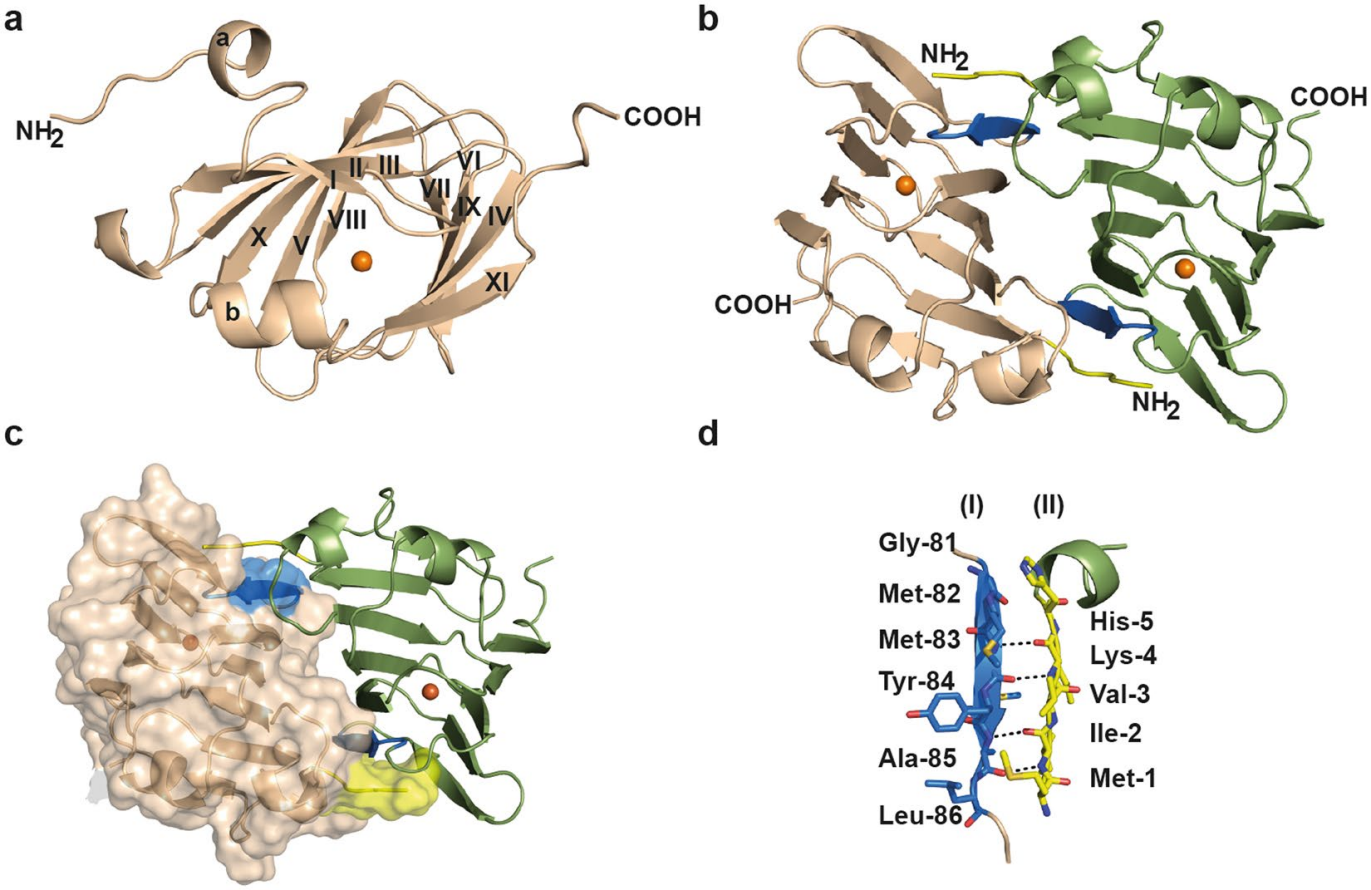
Crystal structure of the (*Pl*)EctC protein and analysis of its dimer interface. The overall fold of the (*Pl*)EctC monomer in its iron-bound state is shown in panel (**a**), and the head-to-tail dimer of the (*Pl*)EctC protein in panel (**b**) as a side-view and the position of the iron in each dimer is indicated by an orange sphere. The dimer interface formed by β-strand 6 of one monomer (shown in blue) and the N-terminal region (shown in yellow) of the other monomer is highlighted. These two figures show the (*Pl*)EctC protein in a cartoon representation. Panel (**c**) represents the interface of the (*Pl*)EctC dimer assembly, where one of the monomers is depicted in a surface representation and the other monomer is shown in a cartoon representation. The areas marked in blue and yellow, respectively, represent the dimer interface, and an orange sphere indicates the position of the iron in each monomer. Panel (**d**) highlights the structural details of the dimer interface with hydrogen bridges between the backbone functional groups of Met-1 to Lys-4 (N-terminal segment of monomer II) with those of Met-83 to Ala 85 (β-strand 6 from monomer I).

The high-resolution dataset obtained for the (*Pl*)EctC::Fe crystal complex was phased using molecular replacement with the crystallographic data of the previously solved structure of the (*Sa*)EctC ectoine synthase (PDB entry 5BXX)^60^ as the search model. After several rounds of model building using COOT^72^ and subsequent refinement, the structure of the full-length (*Pl*)EctC protein was solved (Fig. 3a). This was a substantial improvement over the *S. alaskensis* EctC crystal structure^60^ where the spatial orientation of 22 amino acids from the (*Sa*)EctC COOH-terminus could not be localized in the electron density map, probably due to enhanced structural flexibility of this psychrophilic protein^60,69,73^. The data- and refinement statistics for the (*Pl*)EctC::Fe complex are listed in Table 1.

Once the 1.6 Å structure of the (*Pl*)EctC::Fe protein was completely refined, it was used to phase the other two datasets of the *(Pl)*EctC protein containing iron and either the substrate [*(Pl)*EctC::Fe/*N*-γ-ADABA] or the reaction product [(*Pl)*EctC::Fe/ectoine] of the ectoine synthase. A summary of the data collection statistics, refinement details, and model content of these two (*Pl*)EctC crystal structures is given in Table 1 as well. The (*Pl*)EctC*-Strep*-tag II protein carries an affinity tag attached to its carboxy-terminus; this tag consists of an octapeptide with an additional two amino acid linker (SA-WSHPQFEK) sequence. In the (*Pl)*EctC crystal structures the linker region and five amino acids of *Strep*-tag II sequence was visible, while the last three amino acids were disordered and were therefore not visible in the electron density. The crystallographic data of the three (*Pl*)EctC structures reported in this communication have been deposited in the RCSB Protein Data Bank (https://www.rcsb.org/) under accession number 5ONM [(*Pl*)EctC::Fe], 5ONN [*(Pl)*EctC::Fe/*N*-γ-ADABA], and 5ONO [(*Pl)*EctC::Fe/ectoine], respectively.

The *(Pl)*EctC::Fe/*N*-γ-ADABA crystals diffracted to very high resolution, but they suffered from severe radiation damage, a process, which lowered the diffraction rapidly during data collection. This phenomenon was encountered for more than 20 examined *(Pl)*EctC::Fe/*N*-γ-ADABA crystals. We therefore designed an experiment with a very low radiation dose and exposure time of the crystals to the X-ray beam. This yielded finally a dataset with a resolution of 2.0 Å in which no evidence for radiation damage was observed when the crystallographic data were processed. After manual rebuilding and several cycles of refinement, the *(Pl)*EctC::Fe/*N*-γ-ADABA crystal structure was finalized with an R-factor of 17.1% and an R_*free*_ of 21.7%. The Ramachandran plot revealed that 99.3% of the residues are in the allowed region. Already during the first round of refinement of the *(Pl)*EctC::Fe/*N*-γ-ADABA complex, extra density was visible next to the iron atom, large enough to fit the *N*-γ-ADABA molecule (Fig. S2a). This allowed us to pinpoint and trace the spatial position of the *N*-γ-ADABA substrate within the active site of the ectoine synthase.

Crystals of the (*Pl*)EctC protein in complex with ectoine were obtained after the addition of 20 mM ectoine prior to crystallization trials. Within the cupin fold^63–67^ of the (*Pl*)EctC protein (Fig. 3a), clear density was visible for the ectoine molecule (Fig. S2b), that was subsequently manually placed and included during refinement. After manual rebuilding and several cycles of refinement, the structure of the (*Pl*)EctC::Fe/ectoine complex was finalized with an R-factor of 18.2%, an R_*free*_ of 24%, and a resolution of 2.5 Å. The Ramachandran plot revealed that 99.3% obeyed the rules and fitted into the corresponding plot.

A comparison of the three (*Pl*)EctC crystal structures revealed a root-mean-square deviation (r.m.s.d.) ranging between 0.4 and 0.8 Å over 136 Cα atoms. Keeping in mind that crystal structures are snapshots of the three-dimensional space a given protein can potentially adopt, these data indicate that both the binding of the substrate *N*-γ-ADABA to the EctC protein pre-complexed with iron and the binding of the enzyme reaction product ectoine to the protein do not trigger any gross overall structural changes in the thermo-tolerant (*Pl*)EctC enzyme. A high degree of structural identity was also found between the thermo-tolerant (*Pl*)EctC protein and the cold-tolerant (*Sa*)EctC protein^69,73^; an overall comparison of the three (*Pl*)EctC crystal structures with that of the (*Sa*)EctC protein^60^ revealed r.m.s.d. values ranging between 1.2 and 1.4 Å over 105 Cα atoms. These data indicate that the (*Sa*)EctC and the (*Pl*)EctC ectoine synthases display a highly similar three-dimensional structure despite the fact that these proteins were derived from microorganisms living in ecophysiologically rather different habitats; the effluent of a hot spring (*P. lautus*) and permanently cold ocean waters (*S. alaskensis*)^68,69^.

### Overall fold of the (*Pl*)EctC protein and analysis of the EctC dimer interface

Since the monomers of the three (*Pl*)EctC crystal structures are nearly identical in overall shape, we only describe in the following section the overall structure for the high-resolution (1.6 Å) *(Pl)*EctC::Fe complex in detail. The structure of the (*Pl*)EctC protein consists of 11 β-strands (βI-βXI) and two α-helices (α-a and α-b) (Fig. 3a). The β-strands form two anti-parallel β-sheet regions consisting of βII, βIII, βX, βV, βI, βVI, βIX and βXI. These sets of anti-parallel β-sheets are packed against each other, forming a cup-shaped β-sandwich with a topology characteristic for the widely found cupin-fold of proteins^63–67^.

The structure of the psychrophilic (*Sa*)EctC protein has previously been solved in two different conformations, which were coined the *open* and *semi-closed* states^60^. In the latter state, only part of the carboxy-terminus of the (*Sa*)EctC protein is visible in the electron density map, and it folds into a small helix (α-b) that closes the active site of the enzyme^60^. The formation of the helix α-b induces a reorientation and shift of a long unstructured loop connecting the beta-sheets βIV and βVI of the (*Sa*)EctC protein, resulting in the formation of the stable β-strand βV. In contrast to the (*Sa*)EctC crystal structure, the COOH-terminus of the thermo-tolerant *P. lautus* EctC protein (Fig. 3a) was completely resolved in the electron density map. The remaining segment of the previously unresolved part of the carboxy-terminus of the ectoine synthase from *S. alaskensis* flanks the cupin fold of the (*Pl*)EctC protein and protrudes out of the protein (Fig. 3a).

The (*Pl*)EctC protein is a dimer in solution as revealed by our SEC-MALS analysis (Fig. 1c). Since the asymmetric unit of the (*Pl*)EctC crystal revealed only a monomer, we inspected the crystal packing and analyzed the respective monomer/monomer interactions to elucidate the functional dimer within the crystal structure. The data resulting from this analysis show that the (*Pl*)EctC dimer in the crystal (Fig. 3b) is composed of two monomers arranged in a head-to-tail orientation; it is stabilized via strong interactions mediated by the N-terminus (sequence ^1^MIVKH5) from monomer A and β-strand βVIII from monomer B (sequence ^81^GMMYAL^86^) (Fig. 3b–d). The interactions between these two β-strands rely primarily on backbone contacts (Fig. 3b–d). In addition to these interactions, some weaker hydrophobic interactions between the two monomers are also observed in some loop regions connecting the β-strands; these will probably play more subtle roles in dimer formation. Since the (*Pl*)EctC is a head-to-tail dimer, the interaction interface between the monomers occurs twice in the dimer assembly (Fig. 3b,c). As determined by PISA (Proteins, Interfaces, Structures and Assemblies) analysis^74^, the (*Pl*)EctC monomers interact in the dimer assembly through an extensive surface area of 1501 Å^2^ involving 16 hydrogen bonds and 4 salt bridges. The predicted substantial binding energy of −28.2 kcal mol^−1^ of the two (*Pl*)EctC monomers indicates that these regions represent the predominant interface within the ectoine synthase dimer (Fig. 3b–d).

To identify the structurally closest homologs of the (*Pl*)EctC protein, we performed a DALI search^75^ which recovered, as expected, a variety of cupin-type proteins, most of which are functionally uncharacterized. Not surprisingly, the apo-form of the (*Sa*)EctC protein (PDB accession number: 5BXX)^60^ was found as the structurally closest homolog of (*Pl*)EctC; it had a Z-score of 21.1. Among the proteins with the highest Z-scores recovered by the DALI-search that had been biochemically and functionally studied were the KdgF^76^ and DddK^77^ crystal structures with Z-scores of 13.4 and 13.0, respectively. Like the (*Pl*)EctC protein, the cupin-type KdgF and DddK proteins are dimers that possess an overall topology and a dimer interface very similar to that observed for the ectoine synthase.

The KdgF protein from *Halomonas sp*. is an enzyme that catalyzes a step in the microbial metabolism of uronate sugars from two abundant sources of biomass, pectin and alginate. KdgF mediates the conversion of pectin- and alginate-derived 4,5-unsaturated mono-uronates to form linear ketonized forms^76^. Interestingly, KdgF performs an enzyme reaction (hydrolysis of a cyclic molecule) opposite to that performed by EctC (cyclo-condensation of a linear metabolite). We note in this context that the ectoine synthase from *H. elongata* displays a hydrolytic activity towards synthetic ectoine derivatives with either reduced or expanded ring sizes^70^ but the equilibrium for the EctC-catalyzed *N*-γ-ADABA to ectoine biotransformation lies almost completely on the side of the cyclic condensation product ectoine^36,70^. KdgF exhibits an amino acid sequence identity to (*Pl*)EctC of only 17%, but its crystal structure possesses an r.m.s.d. of 2.1 Å (over 109 Cα atoms) to the ectoine synthase. As determined by PISA^74^, the surface area of the dimer interface of KdgF is 1501 Å^2^, and the monomers interact via 16 hydrogen bonds, and 4 salt bridges, yielding an overall predicted binding energy of the two KdgF monomers of about −30 kcal mol^−1^ ^76^. The crystal structure of KdgF contains a nickel ion, a metal that might have been acquired during the affinity chromatography of the His-tagged recombinant KdgF enzyme purified from *E. coli* cell extracts^76^. As observed with other cupins^63,64,66,67^, the KdgF enzyme is promiscuous with respect to the catalytically required metal, with Co^2+^ being the most effective catalyst among the tested divalent metals^76^.

The DddK protein (PDB accession number: 5TFZ) from the marine bacterium *Pelagibacter ubique* HTCC1062 is a dimethylsulfoniopropionate (DMSP) lyase^77^. This enzyme is involved in the catabolism of the organosulfur compound DMSP, an environmentally abundant organic osmolyte produced by marine algae^78^, yielding the reaction products acrylate and the climate-active gas dimethlysulfide (DMS)^77^. The *P. ubique* DddK protein exhibits an amino acid sequence identity to the (*Pl*)EctC protein of 16.3% and its crystal structure possesses an r.m.s.d. of 1.2 Å (over 126 Cα atoms) to the ectoine synthase from *P. lautus*. The surface area of the dimer interface of DddK^77^ is 1556.1 Å^2^, and the monomers interact with an overall predicted binding energy of −28 kcal mol^−1^. Crystal structures of the DddK protein with either Ni^2+^ or Fe^2+^/Zn^2+^ were recovered^77^, attesting again to the promiscuity of cupins with respect to the metal cofactor used for catalytic activity.

### Structural features of the ectoine synthase and functional relevance of the iron-binding site for enzyme activity

Keeping in mind that the (*Sa*)EctC ectoine synthase is a metal-dependent enzyme, with Fe^2+^ as the physiologically most relevant catalyst^60^, we added Fe(II)Cl_2_ to the (*Pl*)EctC protein solution prior to the crystallization at a final concentration of 4 mM. Clear electron density was visible in the 1.6 Å *(Pl)*EctC crystal structure for an atom with a strong electron density, which cannot be accounted for by a water molecule. To identify the probable nature of this ion, we modeled Mg^2+^, Ca^2+^, Fe^2+^, Ni^2+^, Co^2+^, and Zn^2+^ into the electron density and refined the *(Pl)*EctC crystal structure again. Only when we refined the *(Pl)*EctC structure modeled with Fe^2+^, we observed neither negative nor positive differences in electron density, indicating that iron is indeed the most probable element present in the crystallized *(Pl)*EctC protein.

**Figure 4:**
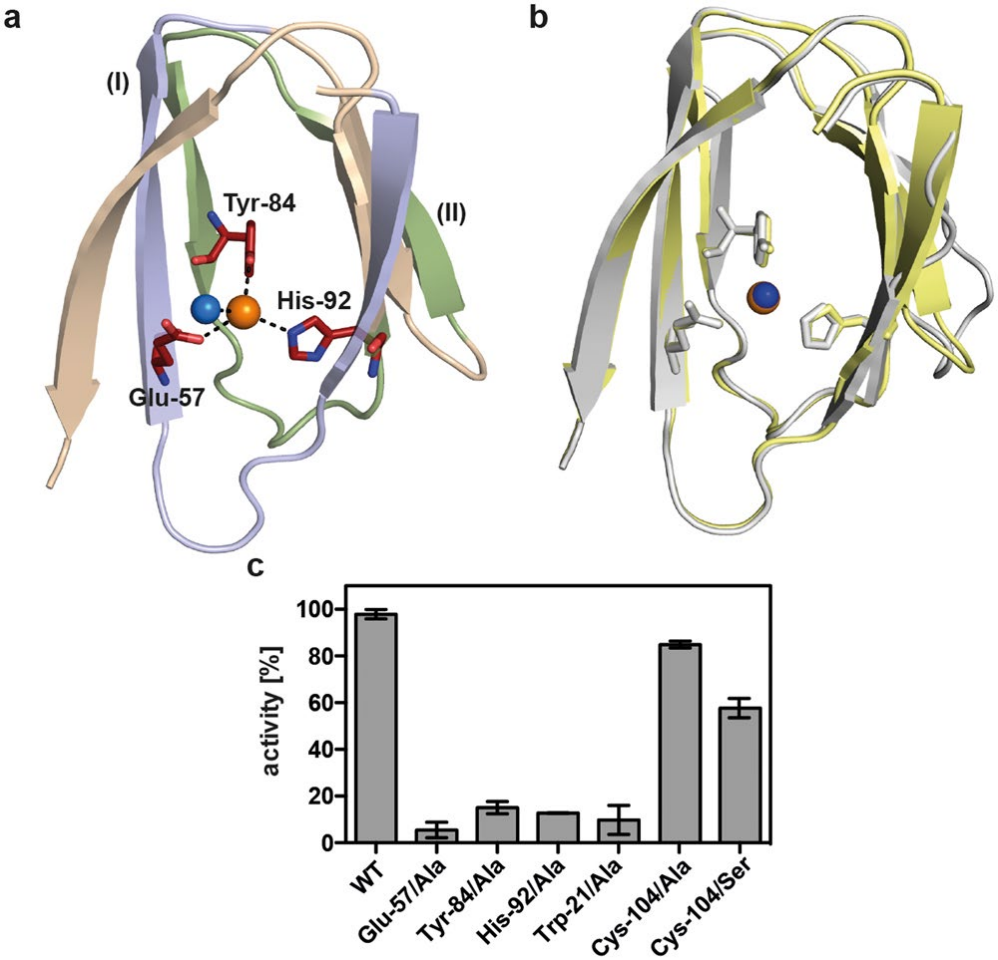
The iron-binding site in the (*Pl*)EctC protein and enzyme activities of selected (*Pl*)EctC variants. (**a**) The iron (represented by an orange sphere) is coordinated by the side-chains of Glu-57, Tyr-84, and His-92 of the (*Pl*)EctC protein with distances of 2.9 Å, 2.8 Å, and 2.9 Å, respectively. The iron-binding site in the substrate-free (*Pl*)EctC crystal structure also contains a localized water molecule (blue sphere); it has a distance of 2.9 Å to the iron atom. The two conserved cupin-motifs include those residues that coordinate the metal ion [G(X)_5_WY(X)_4_**E**(X)_6_G; G(X)_6_PG(X)_2_**Y**(X)_3_G(X)_3_**H**; letters in bold indicate metal-binding residues] are highlighted as part of the overall protein (*Pl*)EctC crystal structure. The first cupin motif [G(X)_5_WY(X)_4_**E**(X)_6_G] is shown in blue, and the second cupin motif [G(X)_6_PG(X)_2_**Y**(X)_3_G(X)_3_**H**] is represented in green. A number of secondary structure elements of the (*Pl*)EctC protein were removed in order to highlight the architecture of the iron-binding site and the position of the two cupin motifs. (**b**) Overlay of the iron-binding site in the (*Pl*)EctC (shown in yellow) and (*Sa*)EctC (shown in grey) crystal structures. The three residues involved in the binding of the Fe(II) ion are depicted as sticks. A water molecule (blue sphere) in the (*Sa*)EctC crystal structure occupies the same location as the Fe(II) ion (orange sphere) in the (*Pl*)EctC structure. (**c**) Single amino acid substitution variants of the (*Pl*)EctC protein were assayed for their enzyme activity. Enzyme activity measurements were conducted under buffer conditions [20 mM HEPES buffer (pH 8.5), 50 mM NaCl, 0.1 mM (NH_4_)_2_Fe(SO_4_)_2_] optimized for the wild-type enzyme using 10 µg of protein and 10 mM of the substrate *N*-γ-ADABA. The enzyme assays were conducted at 30 °C and run for 30 min and the formation of ectoine was monitored by HPLC analysis. The enzyme activity of the mutant (*Pl*)EctC proteins is represented relative to that of the wild-type enzyme (set at 100% activity).

Within the *(Pl)*EctC::Fe crystal structure, the iron atom is tetrahedrally ligated via interactions with the side chains of Glu-57, Tyr-84, and His-92 (Fig. 4a). The distance between the iron atom and the side chains of these three residues are 2.9 Å, 2.8 Å, and 2.9 Å, respectively. A water molecule completes the tetrahedral arrangement of the (*Pl*)EctC iron-binding site in the substrate-free *(Pl)*EctC::Fe crystal structure (Fig. 4a); it is present at a distance of 2.9 Å relative to that of the iron atom. In the previously reported (*Sa*)EctC crystal structure^60^, no metal atom was visible but a water molecule occupied the same position that we observed here for the iron atom in the (*Pl*)EctC crystal structure (Fig. 4b). An overlay of the three iron-coordinating amino acid residues in the (*Pl*)EctC and (*Sa*)EctC^60^ structures revealed that they are perfectly superimposable (Fig. 4b), indicating that (i) the iron-binding site in the ectoine synthase is already preformed in the absence of the catalytically important iron co-factor and that (ii) the binding of the iron atom does not seem to trigger substantial structural rearrangements in the overall fold of the enzyme.

**Figure 5:**
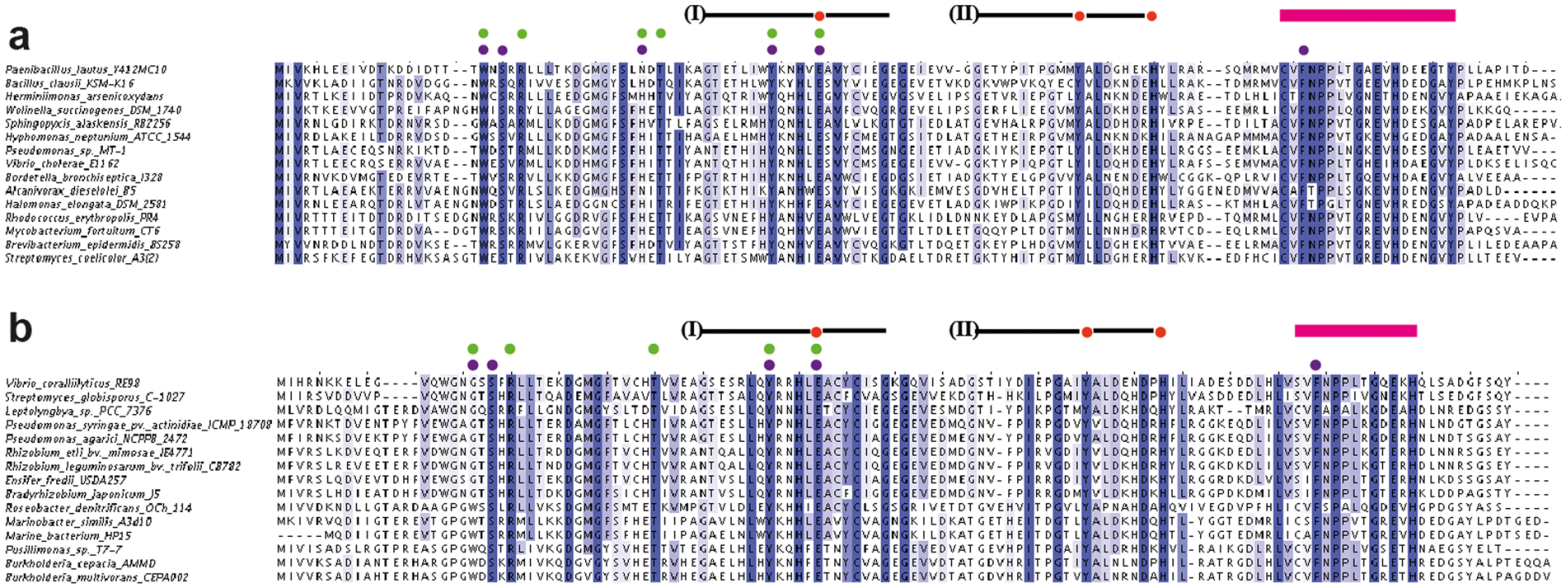
Abbreviated alignment of EctC-type proteins. (**a**) The amino acid sequences of 15 randomly picked EctC proteins from a dataset of 437 ectoine synthases encoded in ectoine biosynthetic gene clusters (*ect*)^5^ were aligned. Strictly conserved amino acid residues are shaded in dark blue. Dots shown above the (*Pl*)EctC protein sequence indicate residues involved in the binding of the iron atom (red), the *N*-γ-ADABA substrate (green), or the enzyme reaction product ectoine (purple). (**b**) The amino acid sequence alignment of 15 EctC-type proteins encoded by orphan *ectC* genes^5^. The same color code shown in panel (**a**) is used to mark those residues that could potentially be involved in iron, substrate, or product binding. The positions of cupin motif-I and cupin motif-II within the EctC amino acid sequence are indicated by a black line. The red bar highlights the region in the C-terminal segment of EctC that forms a lid over the entry to the cupin barrel.

If one considers the architecture of the iron-binding site of (*Pl*)EctC (Fig. 4a) with respect to the previously established consensus sequence for the amino acid sequences involved in metal coordination in cupin-type proteins^63,64,66,67,76,77,79^, both common and distinct features are found. The first consensus cupin motif [G(X)_5_**H**X**H**(X)_3,4_**E**(X)_6_G] is altered in the (*Pl*)EctC protein to G(X)_5_WY(X)_4_**E**(X)_6_G, and the second consensus motif [G(X)_5_PXG(X)_2_**H**(X)_3_N] is changed in (*Pl*)EctC to G(X)_6_PG(X)_2_**Y**(X)_3_G(X)_3_**H** (note: the letters in bold represent those residues that coordinate the metal) (Fig. 5a,b). Thus, in the first consensus cupin motif, none of the two canonical His residues is present in (*Pl*)EctC; however, the canonical Glu residue (Glu-57) is conserved (Fig. 5a,b). In the second cupin motif of the (*Pl*)EctC protein, a Tyr residue (Tyr-84) replaces the canonical His residue, and the motif is elongated to include another His residue (His-92) involved in iron binding (Fig. 4a). Variations in the metal-binding motifs of cupins occur frequently^63,64,66,67,76,77^, but to the best of our knowledge, the one identified here for ectoine synthase is novel (Figs 4a and 5).

In Fig. 4a we have highlighted the positions of the two cupin motifs within the overall (*Pl*)EctC crystal structure, and we point out their position in EctC protein sequences in Fig. 5. By inspecting a recently reported extended amino acid sequence alignment of 582 EctC-type proteins^5^, we found that minor variations in the amino acid sequences of the overall cupin motifs exist, but none of them affects the residues contacting the iron atom directly (Figs 4a and 5). This is highlighted in an abbreviated alignment of amino acid sequences of 15 randomly picked ectoine synthases from the previously reported dataset of 582 EctC-type proteins^5^ (Fig. 5).

The strict evolutionary conservation of the three iron-contacting residues in ectoine synthases attests to their likely critical role for enzyme function. To assess the individual contributions of the (*Pl*)EctC iron-binding residues Glu-57, Tyr-84, and His-92 (Fig. 4a) for the functionality of this enzyme, we replaced each of them individually with an Ala residue via site-directed mutagenesis. The three (*Pl*)EctC variants could be overproduced and purified with the same efficiency as the wild-type (*Pl*)EctC protein. Each of the individual Ala substitution mutations rendered the mutant (*Pl*)EctC proteins in essence catalytically inactive with remaining levels of enzyme activity of 5.4% (Glu-57), 15.1% (Tyr-84), and 12.7% (His-92) (Fig. 4c). Notably, the same conclusion has been reached through mutant analysis of the corresponding putative iron-binding residues present in the (*Sa*)EctC protein^60^.

### Structural features of the binding site for the *N*-γ-ADABA substrate

**Figure 6:**
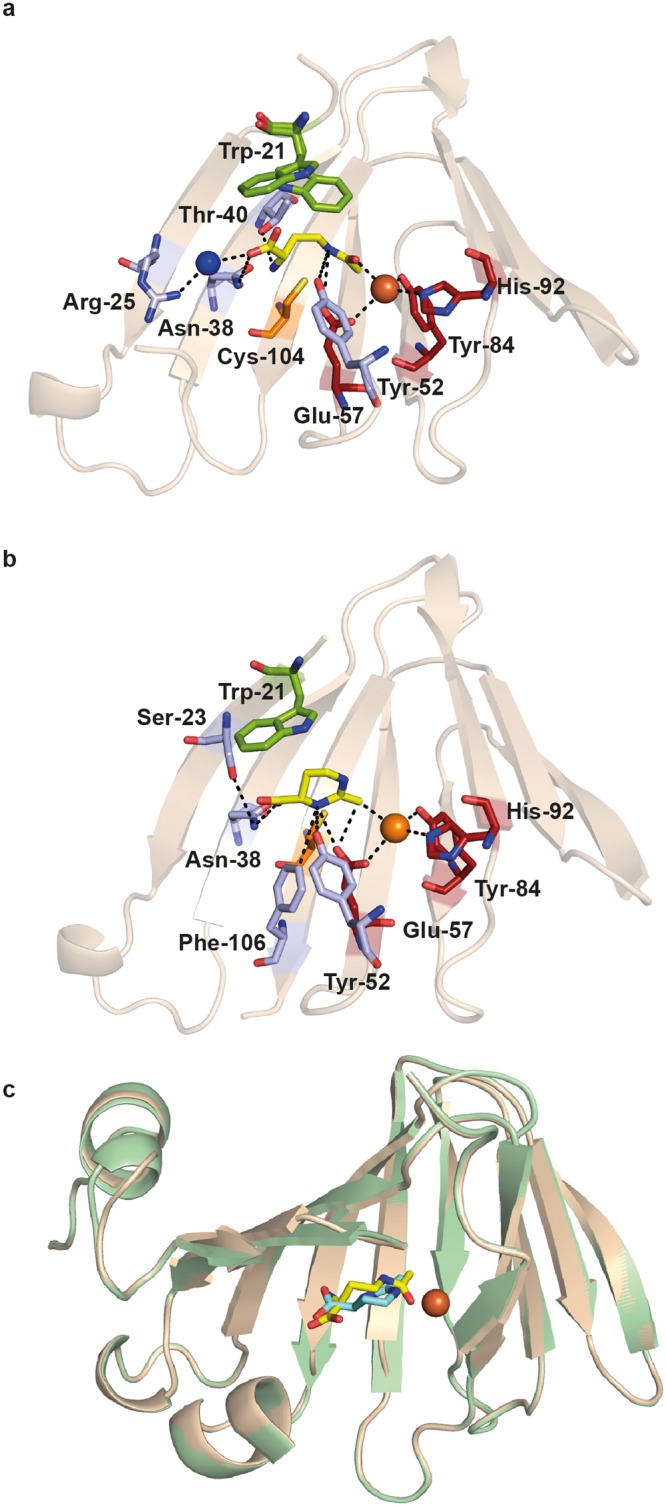
Crystallographic views into the catalytic core of the *P. lautus* ectoine synthase. (**a**) The iron- and substrate-binding network within the catalytic core of the (*Pl*)EctC protein. The side-chain of Trp-21 that adopts two different conformations in the (*Pl*)EctC::Fe/*N*-γ-ADABA crystal structure is emphasized in green. The *N*-γ-ADABA molecule is shown as yellow sticks. The iron is represented as an orange sphere and a water molecule (blues sphere) mediating indirect contacts between the *N*-γ-ADABA molecule and the side-chain of Arg-25 is highlighted. In addition, the spatial position of the side-chain of a conserved Cys residue (Cys-104) close to the active site, but not directly involved in enzyme catalysis is shown. (**b**) Details of the iron- and ectoine-bound state of the (*Pl*)EctC catalytic core are shown. In this structure, the side chain of Trp-21 adopts only a single conformation and provides stabilizing contacts to the ectoine ligand via cation-π interactions. The ectoine molecule is depicted as a yellow stick. (**c**) Cartoon representation of a structural overlay of the (*Pl*)EctC::Fe/*N*-γ-ADABA (gold) and (*Pl*)EctC::Fe/ectoine (green) crystal structures. The position of the substrate and reaction product within the confines of the cupin barrel is highlighted and the iron co-factor is shown as an orange sphere. In all panels, parts of the secondary structure elements of the (*Pl*)EctC protein are omitted in order to provide an unobstructed view into the catalytic core of the ectoine synthase.

In the *(Pl)*EctC::Fe/*N*-γ-ADABA crystal structure, the substrate for the ectoine synthase, *N*-γ-ADABA^36,59^, is positioned in close proximity to the catalytically important iron atom within the cupin barrel (Fig. 6a) and the iron atom is bound in a fashion similar to that observed in the (*Pl*)EctC::Fe complex (Fig. 4a). The substrate *N*-γ-ADABA was added in large excess (40 mM) to the crystallization solution; however, the obtained crystal structure displayed only a partially bound molecule, which resulted in an occupancy of 68% after refinement. The *N*-γ-ADABA molecule is coordinated within the active site of the (*Pl*)EctC enzyme through six direct interactions with residues Trp-21, Arg-25, Asn-38, Thr-40, Tyr-52, and Glu-57. Interactions of *N*-γ-ADABA with the iron atom further stabilize it within the catalytic core of the ectoine synthase (Fig. 6a). In Fig. S3, we provide a numbering scheme for the atoms in the substrate *N*-γ-ADABA and the reaction product ectoine to aid the understanding of the following descriptions.

*N*-γ-ADABA is coordinated in the active site via two sets of interactions. First, a direct interaction occurs between the O atom of the acetyl group of *N*-γ-ADABA (acetamide oxygen) and the iron co-factor with a distance of 2.6 Å. The N5 atom of the substrate interacts with Glu-57 and with Tyr-52; its α-NH_2_ moiety interacts with Thr-40, and one of the carboxylate O atoms interacts with Asn-38. Both of the latter amino acid residues are part of β-sheet βIV. The carboxylate oxygens of *N*-γ-ADABA are also coordinated via interactions with a water molecule, which in turn is held in place via an interaction with the side chain of Arg-25 (Fig. 6a). A second set of interactions is observed for the C3 and C4 atoms of *N*-γ-ADABA, which interact with the side chain of Trp-21 (Fig. 6a). Notably, Trp-21 adopts a dual conformation in the crystal structure of the *(Pl)*EctC::Fe/*N*-γ-ADABA complex, in line with the observed partial occupancy of the crystals with the bound substrate. Only in one of these two conformations of Trp-21, its side chain is oriented towards the *N*-γ-ADABA substrate (52% occupancy) (Fig. 6a). The comparison of the *(Pl)*EctC::Fe and the *(Pl)*EctC::Fe/*N*-γ-ADABA structures therefore suggests that the presence of the *N*-γ-ADABA molecule within the substrate-binding site of the ectoine synthase induces the reorientation of the side-chain of Trp-21 to provide additional stabilizing contacts to the *N*-γ-ADABA molecule. The notion that the side chain of Trp-21 is critically involved in the stable positioning of the substrate in the active site of EctC is supported by data from a site-directed mutagenesis experiment in which we replaced Trp-21 with an Ala residue. This single amino acid substitution yielded a *(Pl)*EctC variant with only 9.7% remaining enzyme activity (Fig. 4c).

A Cys-104/Ala substitution mutation was constructed to assess the importance of this amino acid in enzyme function of the (*Pl*)EctC protein, a residue that is highly conserved in an amino acid sequence alignment of 437 EctC-type proteins encoded by *bona fide ect* gene clusters^5^. Cys-104 is positioned close to the catalytic core, although it appears not to be directly involved in iron binding, or involved in interactions with the substrate *N*-γ-ADABA or the reaction product ectoine within the active site of the ectoine synthase (Fig. 6a,b). Despite the conspicuous spatial arrangement of Cys-104 side-chain (Fig. 6a) and the evolutionary conservation of this residue, the Cys-104/Ala substitution had in essence no effect on the catalytic activity of the (*Pl*)EctC enzyme (Fig. 4c). This result also corroborates the functional relevance of the amino acids whose site-directed change impaired the enzyme function of the (*Pl*)EctC protein (Fig. 4c). Unexpectedly, the replacement of Cys-104 of the (*Pl*)EctC protein by a Ser residue, an amino acid often observed in orphan EctC-type proteins^5,80^ in this amino acid position (Fig. 5b) yielded a (*Pl*)EctC variant that retained only approximately 60% of enzyme activity (Fig. 4c). This drop in enzyme activity is currently not understood in functional terms given the frequent occurrence of a Ser residue at position 104 of ectoine synthases^5,80^.

In the previously reported structural analysis of the *S. alaskensis* EctC protein, it was not possible to obtain (*Sa*)EctC crystals in complex with the natural substrate of the ectoine synthase, *N*-γ-ADABA. Instead, a chemically not fully defined compound (in all likelihood trapped by the recombinant (*Sa*)EctC protein either during purification in *E. coli*, or from the crystallization solution) was present^60^. This C-6 molecule was modeled as hexandiol and it was argued by Widderich *et al*.^60^ that its spatial position within the cupin barrel of the (*Sa*)EctC protein could be used as a proxy for the actual substrate of the ectoine synthase, *N*-γ-ADABA. We now superimposed the *(Pl)*EctC::Fe/*N*-γ-ADABA (PDB accession code: 5ONN) and (*Sa*)EctC/hexandiol (PDB accession code: 5BXX) crystal structures; this yielded a root-mean-square deviation (r.m.s.d.) of 1.4 Å over 105 Cα atoms of the two crystal structures. The overlay showed that the *N*-γ-ADABA and the presumed hexandiol ligand occupy, indeed, a very similar position within the active site of the two studied ectoine synthases (Fig. S4).

### Structural features of ectoine binding within the (*Pl*)EctC active site

We were able to obtain a (*Pl*)EctC crystal structure that contained both the iron ion and an ectoine molecule (Fig. 6b). The ectoine molecule is coordinated within the active site of the (*Pl*)EctC enzyme through five direct interactions with the following residues: Ser-23, Asn-38, Tyr-52, Glu-57, and Phe-106 (Fig. 6b). Ectoine exhibits a significantly different conformation to that of the bound *N*-γ-ADABA molecule. In particular, Tyr-52 and Glu-57 are no longer involved in any H-bonding interactions with N1 of ectoine (derived from the amidic N5 of *N*-γ-ADABA), but now both residues form H-bonds to N3 of ectoine, which is derived from the α-amino group of *N*-γ-ADABA. These new H-bonds towards N3 of ectoine also appear to be stronger than those formed originally with *N*-γ-ADABA, as suggested from their shorter distances (3.2 Å to Tyr-52, and 3.7 Å to Glu-57), and are further stabilized by an interaction with Phe-106 (Fig. 6b). In addition, the side-chain of Asn-38 makes a direct contact to the carboxylate of ectoine, and this side chain is in turn held in place through stabilizing interactions with the side-chain of Ser-23 (Fig. 6b). There may also be a direct interaction between the iron and the methyl group of the ectoine molecule (Fig. 6b). The side chain of Trp-21 adopts a single conformation in the (*Pl*)EctC::Fe-ectoine structure and thereby provides additional stabilizing contacts to the ectoine ligand (compare Fig. 6a,b).

### A structural comparison of the substrate- and product-bound catalytic core of ectoine synthase

When the crystal structures of the *(Pl)*EctC::Fe/*N*-γ-ADABA and the *(Pl)*EctC::Fe/ectoine complexes are overlaid, it becomes apparent that the substrate (*N*-γ-ADABA) and the reaction product (ectoine) occupy almost the same position within the *(Pl)*EctC active site (Fig. 6c). The *(Pl)*EctC::Fe/*N*-γ-ADABA structure reveals an extended conformation of the substrate within the catalytic core (Fig. 6a,c). The distance between the carbonyl C-atom and the α-amino group of the bound *N*-γ-ADABA molecule (4.1 Å) is far too large to allow a direct enzymatic attack to initiate ring closure. As a result, the *N*-γ-ADABA molecule needs to bend significantly in order to correctly position the two substituents of the substrate involved in ring closure (Fig. 1a) closely enough to form the intramolecular Schiff-base bond required to generate the enzyme reaction product ectoine. Hence, the observed mode of ectoine binding indicates the requirement of an extensive conformational change of the linear *N*-γ-ADABA molecule during enzyme catalysis to yield the spatial position of the cyclic ectoine molecule (Fig. 6a–c).

Because the *N*-γ-ADABA molecule is bound in an extended, and not in a pre-bent (Fig. 1a), conformation, the main chain region of *N*-γ-ADABA needs to be rearranged to a more bent conformation during the cyclo-condensation reaction. This requires large movements of the carbon atoms (2.0 Å, 1.2 Å, and 1.4 Å of the C-2, C-3 and C-4 of the *N*-γ-ADABA molecule) to yield the positions of the corresponding C-4, C-5, and C-6 atoms of the resulting ectoine molecule. Moreover, the plane described by the acetamide group of *N*-γ-ADABA indicates a required rotation of about 80° for superimposition with the plane of the amidinium group of ectoine captured in the in the (*Pl*)EctC crystal structure. These changes are associated with an outward spatial extension of the bound *N*-γ-ADABA molecule, which can only occur with an associated flip-over movement of the side chain of Trp-21 from the (partial) position observed in the (*Pl*)EctC::Fe/*N*-γ-ADABA structure (Fig. 6a) to that found in the ectoine-bound structure (Fig. 6b). We note, that the distance between ectoine and the side chain of Trp-21 in the “wrong” conformation (Fig. 6a,b) is only 1.6 Å, a configuration that would likely lead to molecular clashing.

The indole ring of Trp-21 of the (*Pl*)EctC protein stands out as the only amino acid side chain exhibiting a major conformational change between the substrate- and product-bound complexes (Fig. 6a,b). As noted above, this residue is required for enzyme activity (Fig. 4b). Although this residue appears to be present in a mixture of two conformations in the *N*-γ-ADABA bound (*Pl*)EctC structure, we assume that the one deviating from the product-bound state is important for catalysis. In the *N*-γ-ADABA-bound structure, the indole ring of Trp-21 is orientated towards and above the C3 and C4 atoms of the substrate and seems to act as a piston-like element by pushing the *N*-γ-ADABA molecule against the bottom of the active site cavity. This assumption is consistent with the presence of a significant conformational strain in the bound *N*-γ-ADABA molecule, as deduced from large deviations of the bond angles of the C2 and C3 atoms of the diaminobutyrate moiety in the crystal structure from the expected tetrahedral angles (104.9° and 117.5°, respectively, instead of the expected 109.5°). This indicates that Trp-21 might play an important role in exerting conformational constraints on the substrate, which may allow the *N*-γ-ADABA molecule to assume the extended conformation observed in the crystal structure (Fig. 6a). These structural constraints might also move the acetyl group of the substrate close enough towards the iron co-factor to support tautomeric rearrangements of the acetyl substituent.

Apart from the conformational flip of the side-chain of Trp-21 and the reorientation of the bound substrate with respect to the ectoine product, the active site moieties remain remarkably constant in their locations (Fig. 6a,b). Therefore, we assume that the flip-over of Trp-21 triggers the re-orientation of bound substrate during ring formation (or vice versa), and that this coordinated re-organization of the active site is a major driving force for catalysis. Consistent with its predicted critical role in enzyme function (Fig. 4b), we observed that Trp-21 is completely conserved in an amino acid sequence alignment of 437 EctC-type proteins encoded by *ect* gene clusters^5^ (Fig. 5a). The corresponding Trp residue in 145 orphan EctC-type proteins encoded outside canonical *ect* gene clusters^5^ (see below) is conserved as well, but in some cases its position within the EctC protein chain is shifted by two amino acid residues (Fig. 5b).

### Proposal for the ectoine synthase-catalyzed reaction mechanism

**Figure 7:**
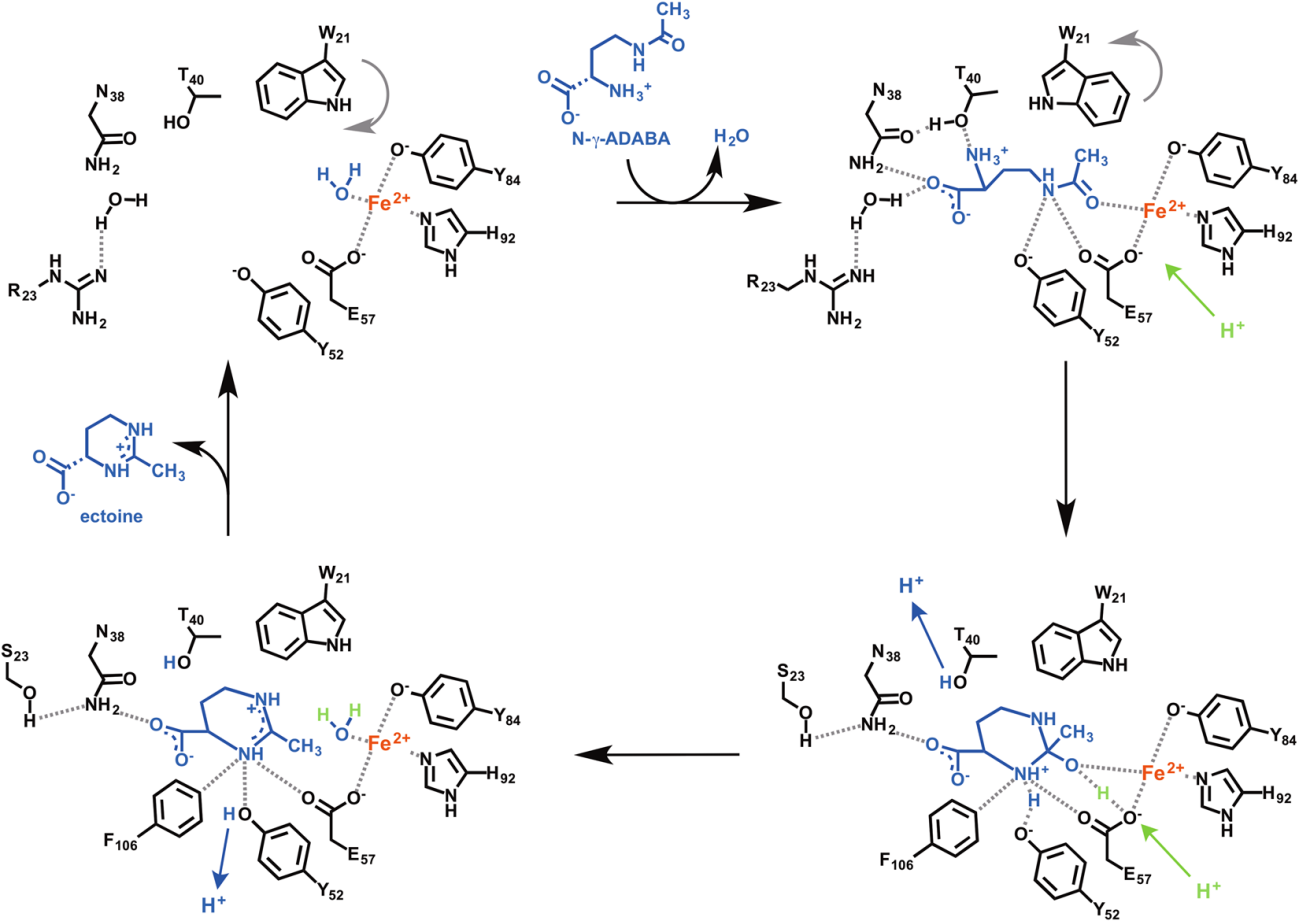
Proposal for the enzyme mechanisms of the ectoine synthase-mediated cyclo-condensation reaction. For a description of the details of the enzyme reaction, see main text.

By comparing the (*Pl*)EctC::Fe to the substrate- and product-bound (*Pl*)EctC structures (Fig. 6a,b), a catalytic cycle for the conversion of the substrate *N*-γ-ADABA into the reaction product ectoine can be suggested (Fig. 7). The (*Pl*)EctC::Fe structure exhibits a tetrahedral coordination of the iron cofactor by the side chains of Glu-57, Tyr-84, His-92 and a water molecule (Fig. 4a). As can be observed in the (*Pl*)EctC::Fe/*N*-γ-ADABA structure, the water is replaced by the amide carbonyl group of *N*-γ-ADABA upon substrate binding (Fig. 6a). This coordination to the Lewis acidic Fe^2+^ cofactor stabilizes the amide functional group of the substrate in its charge-separated mesomeric form with a negative charge at the carbonyl oxygen and a positive charge at the amide nitrogen that is in turn stabilized by cation-π-interactions with the side chain of Trp-21. The resulting increased reactivity of the amide towards nucleophiles triggers the ring closure by attack of the α-amino group of *N*-γ-ADABA that may proceed with a simultaneous proton transfer from the α-amino group or from one of the Fe-coordinating amino acids to the amide carbonyl oxygen. The nucleophilicity of the α-amino group, which is mostly protonated at physiological pH, is increased by the hydrogen bonds to Thr-40 and further to Asn-38 (Fig. 6a,b) thereby allowing a proton transfer during the cyclization of *N*-γ-ADABA to form ectoine. In the next step, the hydroxy group is extruded from the substrate to release the product ectoine (Fig. 7). A simultaneous back-transfer of the proton from Asn-38 via Thr-40 to the expelled hydroxy group may restore the initial binding situation at the central iron with one water ligand (Fig. 7); the product ectoine is finally released from the active site to allow for the initiation of a new catalytic cycle. We note however in this context that the expected water ligand of the Fe^2+^ ion was only confirmed for the substrate-free EctC crystal structure, but not for its ectoine-bound state as this latter crystal structure has a resolution (2.5 Å) that does not allow the positioning of water molecules with confidence.

### The residues forming the entire EctC catalytic core are evolutionarily highly conserved

The explosion in the number of available microbial genome sequences allows one to place the salient features of a given protein within an evolutionary context. We have recently conducted an extensive analysis of the phylogenomics of the ectoine synthase protein family^5^ by relying on the Integrated Microbial Genomes & Microbiomes (IMG/M) database of the DOE’s Joint Genome Institute (JGI) (https://img.jgi.doe.gov/)^81^ for EctC orthologs in members of the *Bacteria* and *Archaea* with deposited complete genome sequences. At the time of the search (13. November 2017), the IMG/M database contained 56 624 bacterial and 1325 archaeal genomes and we identified in this dataset 4493 bacterial and 20 archaeal EctC-type proteins. It should be noted that databases of microbial genome sequences are biased with respect of the type of the represented microorganisms. In our dataset of 4493 bacterial genomes containing *ectC* genes, 1215 *ectC* genes alone were present in the genomes of *Vibrio*-type microorganisms, and 511 *ectC* genes were derived from various *Streptomyces* species and strains^5^. When the phylogenomics of EctC-type proteins was exclusively focused on fully sequenced microbial genomes, 582 predicted EctC-type proteins were retrieved in the search, which were associated with 499 bacterial and 11 archaeal species/strains (Fig. S5)^5^. Hence, in some microbial genomes multiple copies of *ectC*-type genes are present.

Two EctC-type proteins need to be distinguished: (i) those that are encoded in *ect* gene clusters and are thus bona fide ectoine synthases, and (ii) those that are encoded by *ectC*-type genes in microorganisms that either lack *ectAB* genes altogether or contain *ectC*-type gene copie(s) in addition to complete *ectABC* operons^5,60^. A clade analysis of the amino acid sequence of EctC proteins revealed that the EctC proteins encoded by *ect* gene clusters follow, with the notable exceptions of some probable lateral gene transfer events, the taxonomic affiliations of the predicted ectoine-producing microorganisms^5^. However, there is a sub-group of EctC-type proteins that are not part of *ect* biosynthetic gene clusters or that can occur in addition to *bona fide ectC* genes (25% in the dataset examined by Czech *et al*.^5^). A microbial strain (*Pseudomonas syringae* pv. *syringae*) with such an exclusive orphan *ectC* gene has been physiologically studied, and seems to be able to produced ectoine when surface sterilized leaves of the host plant of this pathogen were provided to the culture^80^.

Building on the extensive bioinformatic dataset reported by Czech *et al*.^5^ and on the salient features of the crystal structures of the (*Pl*)EctC protein that we present here (Figs 4 and 6), we now can focus on those ten residues involved in binding iron, the substrate or the product (Fig. 5a,b) in an evolutionary context by inspecting alignments of EctC proteins encoded within complete ectoine biosynthetic gene clusters (437 representatives) and those encoded by orphan *ectC*-type genes (145 representatives). We used the amino acid sequence of the (*Pl*)EctC protein as query for this search. The degree of amino acid sequence identity of EctC proteins encoded by *ect* biosynthetic genes clusters ranged between 90% (for *Paenibacillus gluconolyticus*) and 49% (for *Streptomyces glaucescens*) when 437 amino acid sequences of *bona fide* EctC proteins were aligned and compared with the amino acid sequence of the crystallized (*Pl*)EctC protein. Hence, *bona fide* EctC-type proteins are evolutionarily rather well conserved (Fig. 5a). When the (*Pl*)EctC amino acid sequence was compared with those of 145 orphan EctC-type proteins (Fig. 5b), the degree of amino acid sequence identity decreased and ranged between 42% (*Burkholderia cepacia*) and 37% (*Roseobacter litoralis*).

**Figure 8:**
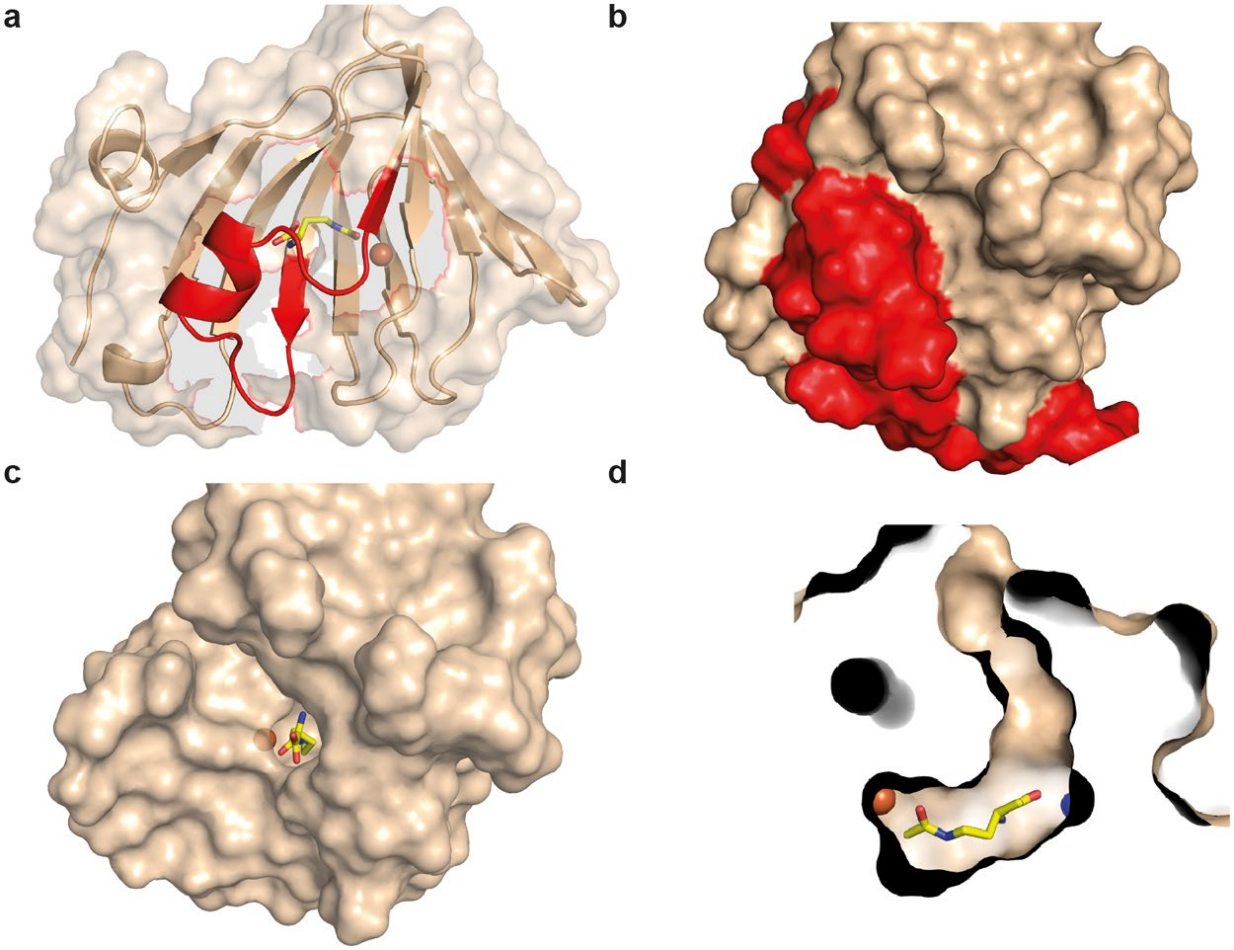
Structural features of the proposed (*Pl*)EctC lid region. (**a**) Ribbon and surface representation of the (*Pl*)EctC::Fe/*N*-γ-ADABA crystal structure highlighting (in red) the lid-region formed by the carboxy-terminal segment of the ectoine synthase. The spatial position of the *N*-γ-ADABA substrate (shown in yellow sticks) and that of the iron atom (shown as an orange sphere) are highlighted within the EctC active site. (**b**) Surface representation of the (*Pl*)EctC::Fe/*N*-γ-ADABA crystal structure in which the lid region is highlighted in red. Relative to the representation of the (*Pl*)EctC crystal structure shown in (a), the crystal structure shown in (b) is slightly rotated in order to provide an expanded view onto the lid region. (**c**) View into the catalytic core of the (*Pl*)EctC::Fe/*N*-γ-ADABA crystal structure after the *in silico* removal of the lid region (amino acids 103 to 130). (**d**) Cross-section through the catalytic core of the (*Pl*)EctC::Fe/*N*-γ-ADABA crystal structure revealing a plausible entry tunnel for the *N*-γ-ADABA substrate (in yellow sticks) and its spatial positions to the catalytically critical iron atom (orange sphere) and a water molecule (blue sphere) that interacts with the *N*-γ-ADABA molecule.

From an alignment of the 582 EctC-type proteins retrieved through the BLAST search of the IMG/M database^5^ we observed that 20 residues were completely conserved. After we excluded the EctC orphan sequences and only compared the 437 EctC-type proteins encoded by *ect* gene clusters, the number of completely conserved amino acid residues increased to 26 (Fig. 5a,b). Based upon the (*Pl*)EctC crystal structures (Fig. 6a,b), these conserved residues can be correlated to the following functions: three residues are involved in metal binding (Glu-57, Tyr-84, His-92), six residues are involved in coordinating the substrate *N*-γ-ADABA within the active site (Trp-21, Arg-25, Asn-38, Thr-40, Tyr-52, Glu-57), and five residues coordinate the reaction product ectoine (Ser-23, Asn-38, Tyr-52, Glu-57, Phe-106). The remaining conserved residues (Fig. 5a) might play either structural, or yet not recognized mechanistic roles. Notably, in the carboxy-terminal segment of EctC proteins, there are nine strictly conserved residues but only one of them (Phe-106) is involved in binding a ligand (ectoine) of the ectoine synthase (Fig. 5a). When one views this carboxy-terminal region in the overall EctC structure, it becomes apparent that it forms a lid over the entry to the cupin barrel (Fig. 8a,b). The strong conservation of the participating residues suggests a functionally important role of this presumed lid region. When one removes the 27 amino acid segment *in silico* from the (*Pl*)EctC::Fe/*N*-γ-ADABA crystal structure, a deep cavity becomes visible that provides a view into the catalytic core of the ectoine synthase with the bound iron, the substrate, and a water molecule (Fig. 8c). These ligands are present at the bottom of a deep tunnel (Fig. 8d).

## Discussion

The last step of the biosynthetic route^36,59^ for the potent microbial stress protectant ectoine^4,5,45^ entails an intramolecular condensation reaction in which the EctA-formed linear *N*-γ-ADABA molecule is cyclized by the reaction of the carbonyl group with the α-amino group, whereby a water molecule is eliminated (Figs 1a and 7). The ectoine synthase (EC 4.2.1.108) mediating this cyclo-condensation reaction is classified as a member of the carbon-oxygen hydro-lyases (EC 4.2.1), but the hydrolytic activity (back reaction) of EctC for its own reaction product ectoine is minimal^36,70^. However, the ectoine synthase from *H. elongata* can hydrolyze, at least to some extent, synthetic ectoine derivatives with either reduced or expanded ring sizes^70^. Judging from the (*Pl*)EctC::Fe-ectoine crystal structure, the cavity of the ectoine synthase active site would be large enough to accommodate the seven-membered ring of the non-natural homoectoine molecule. The *H. elongata* enzyme is also somewhat promiscuous in its biosynthetic activity as it can form the synthetic compatible solute 5-amino-3,4,-dihydro-2H-pyrrole-2-carboxylate (ADPC) through the cyclic condensation of glutamine in a side reaction^70^. Because the EctC-mediated biotransformation of *N*-γ-ADABA into ectoine is practically an irreversible reaction^36,70^, one wonders how it was possible in this study to obtain (*Pl*)EctC::Fe crystals in complex with *N*-γ-ADABA. However, crystallization with this substrate was carried out at pH 4.2, conditions under which the (*Pl*)EctC protein is practically enzymatically inactive (Fig. 2b).

By using an ectoine synthase from the thermo-tolerant bacterium *P. lautus*^68^, we were able to obtain high-resolution crystal structures of the full-length EctC enzyme in complex with its iron-cofactor, its substrate, and its product (Figs 3 and 6). The information obtained from this structural analysis illuminates for the first time the architecture of the catalytic core of ectoine synthase (Fig. 6a–c). The (*Pl*)EctC crystal structures presented here likely represent catalytic states of the enzyme prior and subsequent to catalysis (Figs 3a,b, and 6), thereby providing the foundation for a proposal for the EctC-catalyzed cyclo-condensation reaction (Figs 1a and 7). The ten residues involved in binding of the iron co-factor, the *N*-γ-ADABA substrate, and the reaction product ectoine are evolutionarily highly conserved among a large group of EctC-type proteins^5,30,60^ (Fig. 5a,b). Our structural and functional studies thus render the (*Pl*)EctC protein as a point of reference for the extended ectoine synthase family as a whole^5^ and thereby provide a blueprint for further biochemical and physiological studies.

A previous bioinformatic analysis has indicated that ectoine synthases form a distinct branch within the cupin protein super-family^66,67^. The three-dimensional structure of the ectoine synthase^60^ (and this study) follows the basic design principles of proteins belonging to this super-family^63–67^, and matches closely in its overall fold those of the KdgF and the DddK degradative enzymes. These two cupin-type proteins carry out enzymatic reactions that are different from each other^76,77^ and from that catalyzed by the ectoine synthase (Figs 1a and 7). As expected^66,67^, all residues important for the catalytic activity of the EctC enzyme protrude into the lumen of the cupin barrel (Fig. 6a,b). The (*Pl*)EctC protein is a head-to-tail dimer where backbone interactions between two ß-sheets mediate the contacts between the monomers in the dimer assembly (Fig. 3b–d). A dimeric assembly has previously also been found through biochemical approaches for ectoine synthases from the bacteria *H. elongata*^36^ and *S. alaskensis*^60,73^ and from the archaeon *N. maritimus*^30^, suggesting that dimer-formation is probably a general feature of ectoine synthases.

The catalytically critical Fe^2+^ cation (Fig. 4a) is placed somewhat off-center within the central cavity of the (*Pl*)EctC monomer (Fig. 3a) and is part of an intricate network of interactions that position the *N*-γ-ADABA substrate within the active site of the enzyme close to the metal co-factor (Figs 6a and S4). Variations in the metal-binding motifs of cupins occur frequently^63,64,66,67^, but the one we detected in the ectoine synthase (Figs 4a and 5a,b) is, to the best of our knowledge, novel. Cupins are often promiscuous with respect to the metal used for catalytic activity^76,77,82–84^. For instance, crystals of the DddK DMSP lyase were found in one form to harbor Ni^2+^ while a second crystal form harbored either Fe^2+^ or Zn^2+^ with different levels of occupancy of the same metal binding site^77^. Likewise, although Ni^2+^ was found in the KdgF crystal structure, reconstitution of the metal-depleted recombinant enzyme with various divalent metals identified Co^2+^ as the most effective catalyst^77^. We obtained crystals of the (*Pl*)EctC protein only after adding substantial Fe^2+^ concentrations to the protein solution prior to crystallization and the previously reported crystal structure of the (*Sa*)EctC enzyme lacks a metal altogether^60^. Despite this difference in metal content, the spatial position of the side chains of the three metal-coordinating residues in the active site of the (*Pl*)EctC and (*Sa*)EctC enzymes are super-imposable in their crystal structures (Fig. 4a,b). A metal reconstitution experiment with the (*Sa*)EctC protein has previously revealed that the ectoine synthase can function with various divalent metals (Fe^2+^_,_ Zn^2+^, Co^2+^, Ni^2+^, Cu^2+^, Mn^2+^), albeit with different levels of catalytic activity. Fe^2+^ served as the best-performing co-factor for the (*Sa*)EctC enzyme^60^. Notwithstanding the apparent constraints in interpreting the specific type(s) of metal present in the enzymatically active (*Sa*)EctC^60^ and (*Pl*)EctC proteins, there can be no doubt that the ectoine synthase is metal-dependent. This conclusion is supported by the results of site-directed mutagenesis experiments targeting these residues in both the (*Sa*)EctC^60^ and (*Pl*)EctC proteins (Fig. 4c), and the complete conservation of the three metal-binding residues among the inspected 582 members of the extended EctC protein family^5^ (Fig. 5).

The ligand-binding site for ectoine in the (*Pl*)EctC enzyme differs significantly from those present in ectoine/5-hydroxyectoine-specific extracellular substrate-binding proteins operating in conjunction with either high-affinity ABC- (Ehu)^85^, or TRAP- (Tea and Ueh) type transporters^86,87^. A complex network of interactions with the ectoine molecule is observed in the corresponding EhuB, TeaA, and UehA substrate-binding proteins. It involves several aromatic side-chains, which contribute strongly to high-affinity binding of ectoine with values for the dissociation constant (*K*_d_) of about 1.6 μM, 0.2 μM, and 1.4 μM, respectively^85–87^. In contrast, in the (*Pl*)EctC::Fe-ectoine complex, only the aromatic side chain of Trp-21 contributes to the stabilization of the ectoine molecule in the active site and the carboxylate of ectoine additionally interacts with the side chain of Asn-38 (Fig. 6b).

A key contributor for the co-ordination of the ectoine molecule within the substrate-binding site of the EhuB, TeaA, and UehA ligand-binding proteins is the interaction of the carboxylate of ectoine with the side chain of an Arg residue. Replacement of this Arg residue by Ala abrogates high-affinity ectoine binding^85,87^. While this interaction plays a central role in capturing ectoine stably by the extracellular substrate-binding proteins, it is not observed in the active site of the (*Pl*)EctC cytoplasmic enzyme (Fig. 6b). For ecophysiological reasons, a high substrate affinity is needed to scavenge ectoine through transport processes from scarce environmental sources^88–90^ for its use either as an osmostress protectant^86,91^ or as a nutrient^47,87,92^. On the other hand, a low affinity of the (*Pl*)EctC enzyme for ectoine will be required to release it from the catalytic core once it has been formed by the cyclization of *N*-γ-ADABA (Figs 1a and 7). Hence, the differences observed in the architecture of the ectoine binding sites present in the EhuB, TeaA, and UehA substrate-binding proteins on one hand^85–87^ and the EctC enzyme on the other hand (Fig. 6a,b), most likely reflect functionally imposed constraints on protein structure.

In the previously reported crystal structures of the ectoine synthase from the cold-adapted marine microorganism *S. alaskensis*^69^, the carboxy-terminal segment of the (*Sa*)EctC protein appeared to be highly flexible^60^. However, we have no indications to this effect through the crystallographic snapshots of the (*Pl*)EctC proteins derived from the thermo-tolerant bacterium *P. lautus*^68^. This region (27 amino acids of the 130-amino acids comprising (*Pl*)EctC protein) contains a surprisingly high number of strictly conserved residues among a large group of *bona fide* ectoine synthases (437 representatives) and orphan (145) EctC-type proteins (Fig. 5a,b)^5^. The crystal structures of the (*Sa*)EctC^60^ and (*Pl*)EctC proteins suggest that these conserved carboxy-terminal segments form a lid over the entry to the cupin barrel (Fig. 8a,b). The catalytic core of the ectoine synthase is positioned at a bottom of a deep tunnel (Fig. 8d), and one can therefore readily envision that movement of the lid provides access of the *N*-γ-ADABA substrate to the active site (Figs 3a and 6) and a subsequent exit route for the reaction product ectoine. It therefore seems plausible that the lid region shields the active site of the ectoine synthase from the external solvent and thereby provides a privileged space for the elimination of a water molecule during the cyclo-condensation enzyme reaction (Figs 1a and 7). Moreover, opening and closing of the lid domain may also be required for the incorporation of the catalytically important metal ion into the active site.

When one views the EctC protein family in a phylogenomic context^30,60^, one finds that the EctC biosynthetic enzyme is taxonomically affiliated with ten bacterial (including five sub-phyla of the *Proteobacteria*) and two archaeal phyla^5^ (Fig. S5). The ability to produce the stress protectant ectoine is thus primarily a trait of the *Bacteria*^30^, with a dominant representation of ectoine producers among members of the *Proteobacteria* and *Actinobacteria*^5^. The few *Archaea* that possess *ect* biosynthetic genes (Fig. S5) have likely acquired them from bacterial donors via lateral gene transfer^30^. Such genetic events also seem to be responsible for the introduction of ectoine biosynthetic genes into a few halophilic bacteriovorous protists^27,28^, as these unicellular *Eukarya* might have gained the *ect* biosynthetic genes from ectoine-producing food bacteria living in the same high-saline habitat^29^.

A previous study^60^ and our recent comprehensive phylogenomic analysis^5^ revealed the existence of a substantial group of EctC-type proteins that are mostly found in a taxonomically rather heterogeneous group of microorganisms lacking canonical *ect* biosynthetic operons (Fig. S5). The physiological and biochemical function of these orphan EctC-type proteins is not yet clear^5,60,80^. They may either be remnants of originally functional ectoine biosynthetic gene clusters, or they may possess catalytic activities that might rely on a substrate chemically related in its structure to *N*-γ-ADABA. The orphan EctC proteins often differ notably in their amino acid sequence from that of EctC proteins encoded by genes present in the complete *ectABC* gene cluster (Fig. 5a,b). However, when these proteins are now viewed within the framework of the (*Pl*)EctC crystal structures, we found that their predicted iron-, *N*-γ-ADABA- and ectoine-binding residues are mostly conserved, as is the spatial relationship of these residues within the main amino acid chain (Fig. 5a,b). Hence, the orphan EctC proteins possess the structural hallmarks of ectoine synthases, as suggested by an exploratory physiological study with the plant pathogen *P. syringae* pv. *syringae*^80^. We do not know, however, of any anabolic or catabolic process in microorganisms that would yield the metabolite *N*-γ-ADABA except in the context of ectoine biosynthesis^36,59^ and, perhaps, also as the result of ectoine degradation^47,92,93^. Our insights into the structure/function relationship of *bona fide* ectoine synthase reported here and the presented physiological and phylogenomic considerations (Fig. S5) might therefore serve as primers to study the substantial group of orphan EctC-type proteins^5^, both biochemically and structurally in the future to reveal their physiological function.

## Methods

### Chemicals

Ectoine was a kind gift from the bitop AG (Witten, Germany). Anhydrotetracycline hydrochloride (AHT), desthiobiotin, and Strep-Tactin Superflow chromatography material for the purification of *Strep*-tag II labeled proteins were purchased from IBA GmbH (Göttingen, Germany). The commercially unavailable substrate for the ectoine synthase, *N*-γ-acetyl-L-2,4-diaminobutyric acid (N-γ-ADABA), was synthesized through alkaline hydrolysis of ectoine^94^. It was purified from the byproduct *N*-α-acetyl-L-2,4-diaminobutyric acid (N-α-ADABA) by repeated chromatography on a silica gel column (Merck silica gel 60) using a gradient of ethanol/25% ammonia/water 50:1:2–10:1:2 as eluent^60^. The identity and purity of the isolated *N*-γ-ADABA was monitored by thin-layer chromatography and nuclear magnetic resonance spectroscopy (^1^H-NMR and ^13^C-NMR) on a Bruker AVIII-400 or DRX-500 NMR spectrometer as described^59,60,94^. All chemicals used to synthesize and purify *N*-γ-ADABA were purchased from either Sigma Aldrich (Steinheim, Germany) or Acros (Geel, Belgium). Numbering of the *N*-γ-ADABA and ectoine follows the IUPAC rules, applying replacement nomenclature for *N*-γ-ADABA as “2-amino-5-aza-6-oxoheptanoic acid” (see Fig. S3).

### Recombinant DNA procedures and construction of plasmids

The DNA sequences of the *ectC* genes from the genome sequence of *P. lautus* Y4.12MC10 (accession number: NC_013406)^68^ was used as the template for the synthesis of a codon-optimized version of *ectC* (LifeTechnologies; Darmstadt, Germany) for its heterologous expression in *E. coli*. The DNA sequence of this synthetic *ectC* gene has been deposited in the NCBI database under accession number KR002038. To allow the overproduction of the recombinant *(Pl*)EctC protein in *E. coli* and its purification via affinity chromatography, an overexpression plasmid was constructed, which directs the synthesis of a (*Pl*)EctC-*Strep*-tag II recombinant protein. For this purpose, the *ectC*-containing DNA fragment was retrieved from the plasmid provided by the supplier of the synthetic construct (LifeTechnologies), and inserted into the expression vector pASG-IBA3 (IBA GmbH, Göttingen, Germany). The *ectC* coding sequence was cloned into plasmid pASG-IBA3 in such a way that the resulting EctC protein is fused at its carboxy-terminus to a *Strep*-tag II affinity peptide (SA-WSHPQFEK), thereby allowing the purification of the (*Pl*)EctC-*Strep*-tag II protein by affinity chromatography on a streptavidin affinity matrix (IBA GmbH, Göttingen, Germany). The transcription of the *ectC* gene in the resulting plasmid pWN14 [*P. lautus ectC*^+^; constructed by N. Widderich] is mediated by the *tet* promoter present on the backbone of the expression vector pASG-IBA3 and controlled through the TetR repressor whose DNA-binding activity can be abrogated by adding the synthetic inducer AHT to the growth medium.

Variants of the codon-optimized *ectC* gene from *P. lautus* present on plasmid pWN14 were prepared by site-directed mutagenesis using the Q5 Site-Directed Mutagenesis Kit (New England BioLabs GmbH, Frankfurt a. M., Germany) with custom synthesized DNA primers purchased from Microsynth AG (Lindau, Germany). The DNA sequence of the entire coding region of each mutant *ectC* gene was determined by Eurofins MWG (Ebersberg, Germany) to ensure the presence of the desired mutation and the absence of unwanted alterations. The following variants of the *P. lautus ectC* gene were constructed: pLC55 (Glu-57/Ala; GAA/GCA), pLC56 (Tyr-84/Ala; TAT/GCT), pLC57 (His-92/Ala; CAT/GCT, pLC58 (Cys-104/Ala; TGT/GCT), pLC59 (Cys-104/Ser; TGT/TCT), and pLC67 (Trp-21/Ala; TGG/GCG).

### Bacterial strains, media, and growth conditions

The *E. coli* strain TOP10 (Invitrogen, Carlsbad, CA, USA) was used for the propagation of plasmids carrying *ectC* genes. Cultures of the plasmid-carrying *E. coli* strain were grown at 37 °C in Luria-Bertani (LB) liquid medium containing ampicillin (100 µg ml^−1^). Heterologous overproduction of the plasmid-encoded *P. lautus* EctC *Strep*-tag II protein [(*Pl*)EctC] was carried out in the *E. coli* B strain BL21 in modified minimal medium A (MMA)^95^ containing 0.5% (w/v) glucose as the carbon source and 0.5% (w/v) casamino acids, 1 mM MgSO_4_, and 3 mM thiamine as supplements. Mutant derivatives of the (*Pl*)EctC protein were overproduced and purified as described below for the corresponding wild-type proteins.

### Overproduction, purification and analysis of the quaternary assembly of EctC proteins

Cells of the *E. coli* B strain BL21 harboring plasmid pWN14 (*ectC*^+^) were inoculated into modified MMA (1 L medium in a 2-L Erlenmeyer flask) to an OD_578_ of 0.05 from an overnight culture. The cells were grown on an aerial shaker (set to 180 rpm) at 37 °C until the cultures reached an OD_578_ of 0.5. At this time point the synthetic inducer AHT for the TetR repressor was added to a final concentration of 0.2 mg ml^−1^ to trigger enhanced transcriptional activity of the *tet* promoter and thereby boost the expression of the plasmid-encoded *ectC* gene. After 2 h of further growth of the culture at 37 °C, the *E. coli* BL21 (pWN14) cells were harvested by centrifugation (4 600 × g) and disrupted by passing them through a French Pressure cell (at 1 000 psi); a cleared cell lysate was prepared from these disrupted cells by ultracentrifugation (100 000 × g) at 4 °C for 45 min as described^73^. Cleared cell extracts of the (*Pl*)EctC*-Strep*-tag II overproducing cultures were used to purify the recombinant proteins by affinity chromatography on Strep-Tactin affinity resin as detailed previously^73,96^. The concentration of the (*Pl*)EctC protein in the individual fractions eluted from the Strep-Tactin Superflow affinity column was measured with the Pierce BCA Protein Assay Kit (Thermo Fisher Scientific, Schwerte, Germany). The purity and apparent molecular mass of the (*Pl*)EctC protein was assessed by SDS–PAGE (15% polyacrylamide), and the PageRuler Prestained Protein Ladder (Thermo Fisher Scientific) was used as a reference to assess the electrophoretic mobility of the (*Pl*)EctC*-Strep*-tag II protein. The recombinant (*Pl*)EctC protein was concentrated to approximately 10 mg ml^−1^ with Vivaspin 6 columns (Sartorius Stedim Biotech, Göttingen, Germany) with a 10-kDa molecular-weight cutoff value prior to crystallization trials.

To analyze the quaternary assembly of the (*Pl*)EctC protein, we used high-performance liquid chromatography coupled to multi-angle light scattering detection (HPLC-MALS). For these experiments, an Agilent Technologies system connected to a triple-angle light scattering detector (miniDAWN TREOS, Wyatt Technology Europe GmbH, Dernbach, Germany) followed by a differential refractive index detection system (Wyatt Technology) was used. Typically, 200 µl of purified (*Pl*)EctC protein (2 mg ml^−1^) was loaded onto the Bio SEC-5 HPLC column and the obtained data were analyzed with the ASTRA software package (Wyatt Technology).

### Ectoine synthase enzyme activity assay

Ectoine synthase activity of the (*Pl*)EctC protein was determined by HPLC-based enzyme assays^30,60^. The EctC-mediated conversion of *N*-γ-ADABA into ectoine was performed in a 30-µl reaction volume containing 10 mM *N*-γ-ADABA, 0.1 mM (NH_4_)_2_Fe(SO_4_)_2_, 20 mM HEPES (pH 8.5), and 50 mM NaCl at a temperature of 30 °C in a water bath. For these reactions 1 µg of purified EctC protein was used. Each enzyme assay was run for 2.5 min and was stopped by adding 30 µl of acetonitrile (100%) to the reaction vessel. The samples were then centrifuged (16 060 × g, at room temperature for 10 min) to remove the denatured proteins and the supernatant was subsequently analyzed for the formation of ectoine by HPLC analysis. 10-μl samples were injected into the HPLC system and chromatographed through a GROM-SIL Amino-1PR column (125 × 4 mm with a particle size of 3 µm) that was purchased from GROM (Rottenburg-Hailfingen, Germany). The amounts of the EctC-catalyzed enzyme reaction product ectoine in individual samples was monitored using an Infinity 1260 Diode Array Detector (DAD) (Agilent, Waldbronn, Germany) at 210 nm integrated into an Agilent 1260 Infinity LC system (Agilent). The ectoine content of the samples was quantified using the OpenLAB software suite (Agilent) using commercially available ectoine (bitop AG, Witten, Germany) as the standard. For the ectoine synthase enzyme activity assays, three independently isolated (*Pl*)EctC protein preparations were used and each data-point from the individual protein preparations was assayed twice.

When mutant (*Pl*)EctC proteins were assayed for their enzyme activity, the buffer conditions optimized for the wild-type enzyme were used, but 10 µg of purified (*Pl*)EctC protein were employed and the reaction time was extended to 30 min. During the initial screening for the pH optimum of the (*Pl*)EctC enzyme, a buffer mixture of MES (pH 5.5), PIPES (pH 6.5), TES (pH 7.5), CHES (pH 8), HEPES (pH 8.5), and CAPS (pH 10) (20 mM each) was used. The pH values of these buffer solutions were adjusted with 38% HCl or 5 M NaOH at a temperature (30 °C) that was also used for the ectoine synthase enzyme reaction.

### Crystallization of the (*Pl*)EctC protein

Crystal screening was carried out at 285 K using the sitting-drop vapor-diffusion method. Several initial crystallization conditions for the (*Pl)*EctC-*Strep*-tag II protein were obtained using commercial screens from NeXtal (Qiagen, Hilden, Germany) and Molecular Dimensions (Suffolk, England) in Corning 3553 plates. The homogenous (*Pl)*EctC protein solution (8–12 mg ml^−1^ in 20 mM Tris, pH 7.5, 200 mM NaCl) was premixed first with 100 mM Fe(II)Cl_2_ to a final concentration of 4 mM and subsequently with either 1 M ectoine (to a final concentration of 40 mM) or 500 mM *N*-γ-ADABA (to a final concentration of 20 mM). These protein solutions were incubated on ice for one hour prior to crystallization trials. In these crystallization trials, 0.1 µl (*Pl)*EctC protein solution was mixed with 0.1 µl reservoir solution and equilibrated against 50 µl reservoir solution. (*Pl)*EctC crystals were formed under several conditions, and the most promising one consisted of 0.2 M ammonium sulfate, 0.1 M phosphate citrate (pH 4.2), 20% (v/v) PEG 300, 10% (v/v) glycerol from NeXtal Core II suite (Qiagen, Hilden, Germany). The first crystals were obtained after around twelve hours and reached the maximum dimensions of about 120 × 45 × 30 µm^3^ (with ectoine) and 250 × 45 × 35 µm^3^ (with *N*-γ-ADABA). The crystallization conditions were optimized by grid screens around the initial condition and by variation of the combination of the added substrates. The drops of *(Pl)*EctC protein composed of 1 µl protein solution and 1 µl reservoir solution were equilibrated against 300 µl reservoir solution in sitting drops. Different premixes were set up (final concentrations): (i) 4 mM Fe(II)Cl_2_, (ii) 40 mM ectoine, (iii) 20 mM *N*-γ-ADABA, (iv) 4 mM Fe(II)Cl_2_, 40 mM ectoine, or (v) 4 mM Fe(II)Cl_2_, 20 mM *N*-γ-ADABA. Large crystals were obtained after twelve hours either without any substrate or with ectoine, *N*-γ-ADABA, iron or alternatively the combination of Fe(II)Cl_2_ and ectoine or Fe(II)Cl_2_ and *N*-γ-ADABA. The largest crystals reached dimensions of 500 × 200 × 100 µm^3^. All crystals were cryoprotected by carefully overlaying the crystallization drop with 3-µl mineral oil before the crystals were harvested and flash-frozen in liquid nitrogen.

### Data processing and structure determination

Data sets were collected from a single crystal of either *(Pl)*EctC::Fe, *(Pl)*EctC::Fe/*N*-γ-ADABA, and EctC::Fe/ectoine on beamline P13 at DESY (EMBL, Hamburg, Germany) and/or ID29 at the ESRF, Grenoble, France at 100 K. These data sets were processed using the XDS package^97^ and scaled with XSCALE^98^. Initial phases were obtained by molecular replacement using the program PHASER^72^ with the crystal structure of the *S. alaskensis* EctC protein (PDB entry 5BXX) (without taking its side chains into account) as a template^60^. Model building and refinement were performed using COOT^99^ and REFMAC5^100^. Data refinement statistics and model content are summarized in Table 1. The atomic coordinates and structure factors have been deposited in the Worldwide Protein Data Bank (PDB) (https://www.wwpdb.org/) under the following accession codes: for the *(Pl)*EctC::Fe complex, 5ONM for the *(Pl)*EctC::Fe/*N*-γ-ADABA complex, and 5ONO for *Pl)*EctC::Fe/ectoine complex.

### Figure preparation of crystal structures

Figures of the crystal structures of the (*Pl)*EctC protein were prepared using the PyMol software suite (www.pymol.org)^101^.

### Database searches and phylogenetic analysis of EctC-type proteins

The amino acid sequence of the *P. lautus* EctC protein (accession number: YP_003245677) was used as the template for BLAST searches^102^ (of all finished sequences of the microbial database of the US Department of Energy Joint Genome Institute (http://jgi.doe.gov/)^81^. EctC-type amino acid sequences^5^ were compared using the MAFFT multiple amino acid sequence alignment server (http://mafft.cbrc.jp/alignment/server/)^103^. This data set was then used to construct a rooted phylogenetic tree of EctC-type sequences^5^ by employing the iTOL software suit (http://itol.embl.de/)^104^. The dimer interface of the (*Pl*)EctC protein was analyzed using PISA^74^ (http://www.ebi.ac.uk/pdbe/pisa/). Structural homologs of (*Pl)*EctC were searched using the DALI-web server (http://ekhidna.biocenter.helsinki.fi/dali_server/start)^75^ using the (*Pl*)EctC::Fe crystal structure as the search query.

## Electronic supplementary material

Supplementary data

## Data Availability

All data generated or analyzed during this study are included in this published article (and in its accompanying Supplementary Information). The atomic coordinates and structure factors for the crystal structures of the (*Pl)*EctC protein determined in this study have been deposited in the Protein Data Bank with accession codes 5ONM for the *(Pl)*EctC::Fe complex, 5ONN for the *(Pl)*EctC::Fe/*N*-γ-ADABA complex, and 5ONO for *Pl)*EctC::Fe/ectoine complex.
