# Supplementary material for: Illuminating the catalytic core of ectoine synthase through structural and biochemical analysis
Source: Sci Rep. 2019 Jan 23;9:364. doi: 10.1038/s41598-018-36247-w (PMC6344544; doi:10.1038/s41598-018-36247-w)
Supplement: Supplementary file 1 — Supplementary data [file 41598_2018_36247_MOESM1_ESM.pdf]

Supplementary Data

Illuminating the catalytic core of ectoine synthase through structural and biochemical analysis

Laura Czech[1¶], Astrid Höppner[2,¶], Stefanie Kobus[2], Andreas Seubert[3], Ramona Riclea[4,], Jeroen S. Dickschat[4,], Johann Heider[1,5], Sander H.J. Smits[2,6,*], and Erhard Bremer[1,5,*]

[1]Department of Biology, Laboratory for Microbiology, Philipps-University Marburg, D-35043 Marburg, Germany
[2]Center for Structural Studies, Heinrich-Heine-University Düsseldorf, D-40225 Düsseldorf, Germany
[3]Department of Chemistry, Analytical Chemistry, Philipps-University Marburg, D-35043 Marburg, Germany
[4]Kekulé-Institute for Organic Chemistry and Biochemistry, Friedrich-Wilhelms-University Bonn, D-53121 Bonn, Germany
[5]LOEWE Center for Synthetic Microbiology, Philipps-University Marburg, D-35043 Marburg, Germany
[6]Institute of Biochemistry, Heinrich-Heine-University Düsseldorf, D-40225 Düsseldorf, Germany

[¶]Both authors contributed equally

*co-corresponding authors

Running title: Crystal Structure of Ectoine Synthase

Please direct all correspondence concerning this manuscript **during the reviewing, editorial and printing process to Dr. Erhard Bremer.** Philipps-University Marburg, Dept. of Biology, Laboratory for Microbiology, Karl-von-Frisch-Str. 8, D-35032 Marburg, Germany. Phone: (+49)-6421-2821529. Fax: (+49)-6421-2828979. E-Mail: bremer@staff.uni-marburg.de

Correspondence to Sander H.J. Smits: Institute of Biochemistry, Heinrich-Heine-University Düsseldorf, Universitätsstrasse 1, D-40225 Düsseldorf, Germany. Phone: (+49)-2118-112647. Fax: (+49)-211-8115310. E-Mail: sander.smits@hhu.de

Correspondence to Erhard Bremer: Department of Biology, Laboratory for Microbiology, Philipps-University Marburg, Karl-von-Frisch Strasse 8, D-35043 Marburg, Germany. Phone: (+49)-6421-2821529. Fax: (+49)-6421-2828979. E-Mail: bremer@staff.uni-marburg.de

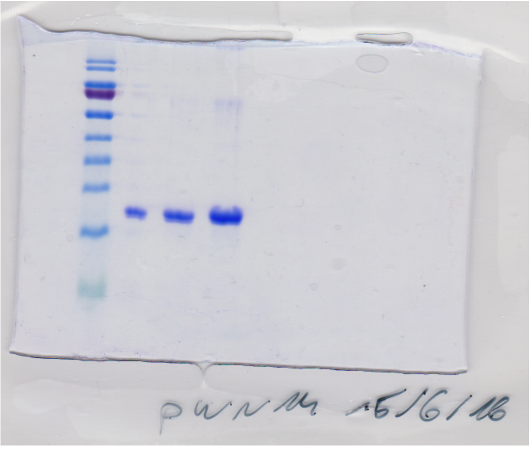

Supplementary Figure S1 Original unprocessed version of the SDS gel included in Figure 1b of the main text.

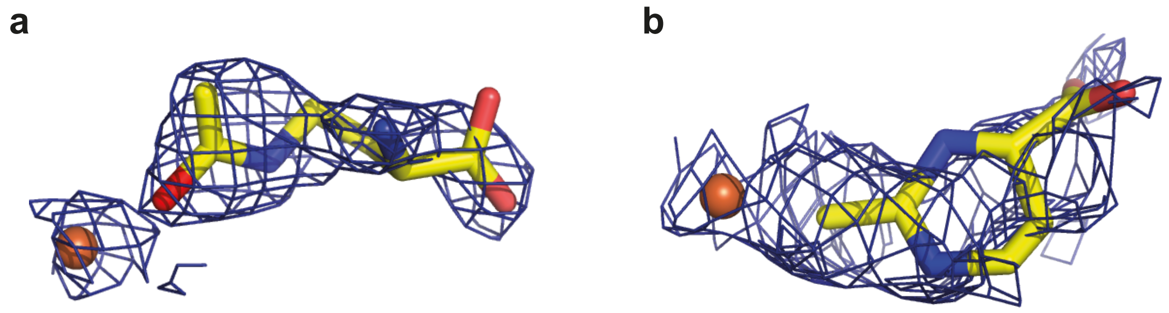

Supplementary Figure S2 Electron densities of (a) the substrate *N*-γ-ADABA derived from the (*Pl*)EctC::Fe/*N*-γ-ADABA complex (PDB code: 5ONN) and of (b) the EctC-generated enzyme reaction product ectoine derived from the (*Pl*)EctC::Fe/ectoine crystal structure (PDB code: 5ONO). The iron atom crucial for the EctC-mediated cyclo-condensation reaction of the linear *N*-γ-ADABA substrate molecule to form the cyclic ectoine reaction product, is shown as an orange sphere. The electron densities around the *N*-γ-ADABA and ectoine molecules and the iron atom are contoured at 1.0 sigma. The figures were prepared using Phenix and Pymol[1,2].

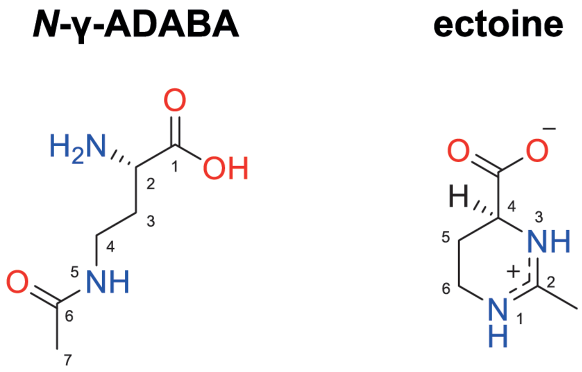

Supplementary Figure S3 Numbering of the C- and N- atoms of the substrate *N*-γ-ADABA and the product ectoine of the ectoine synthase EctC.

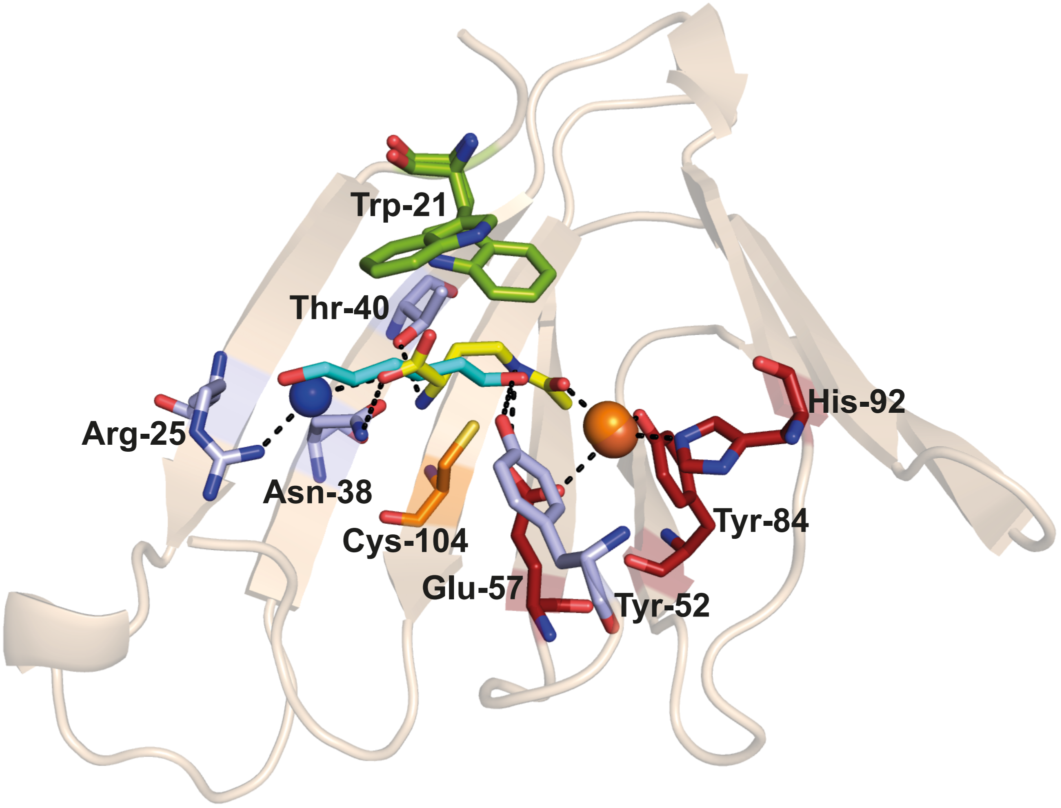

Supplementary Figure S4 Overlay of the substrate *N*-γ-ADABA (depicted in yellow) bound in the (*Pl*)EctC crystal structure (PDB code: 5ONN) and the proposed hexandiol ligand (shown in blue) found in the catalytic core of the previously reported (*Sa*)EctC crystal structure (PDB code: 5BXX)[3]. The superimposition of the *(Pl)*EctC::Fe/*N*-γ-ADABA and (*Sa*)EctC/hexandiol complexes revealed a root-mean-square deviation (r.m.s.d.) of 1.4 Å over 105 Cα atoms of the two crystal structures.

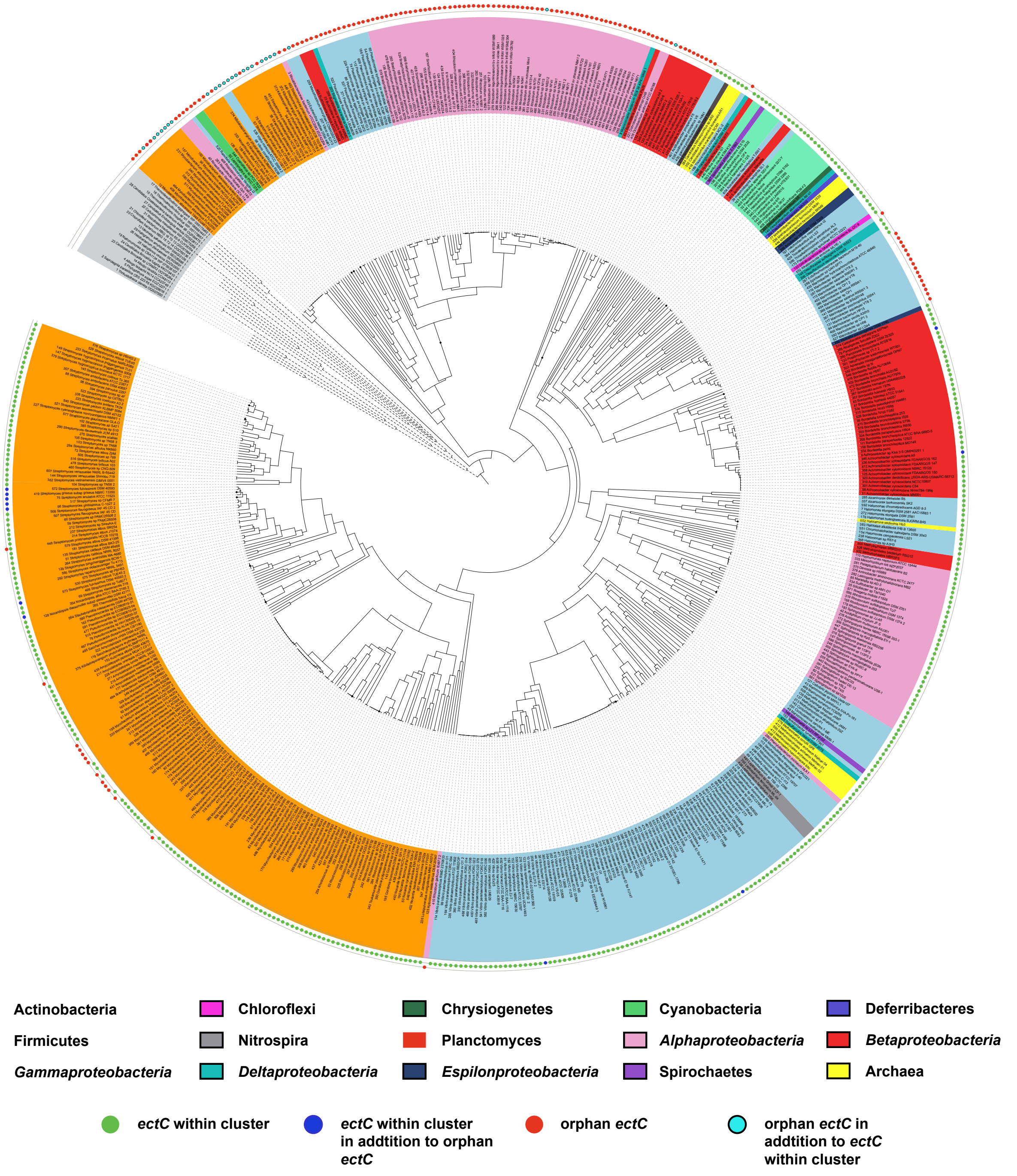

Supplementary Figure S5 Phylogenomics of the ectoine synthase. The amino acid sequences of 582 EctC-type proteins were retrieved from microorganisms with fully sequenced genomes, aligned using MAFFT [4] and then used for a clade analysis with the iTOL software[5]. The tree was rooted with a number of microbial proteins that belong to the cupin-superfamily[6,7] to which the EctC protein also belongs[3]. The phylogenetic affiliation of the various EctC proteins is depicted in different colors shown in the outer ring, and the color code is explained in the figure. The dots in the outmost rings depict (from the inside to the

outside) if the EctC protein is encoded within an *ect* biosynthetic gene cluster or if the EctC-type protein is an orphan. This figure was modified from the data compiled and published in a previous study[8].

Supplementary Table S1 DNA primers used in this study for the introduction of site directed mutations into the codon-optimized *P. lautus* gene.

**Plasmid**	**Mutation**	**Primer name**	**Primer sequence**[a]
pLC55	Glu-57/Ala	(Pl)EctC_E57A_for	AACCATGTTG**C**AGCCGTGTATTG
	GAA → GCA	(Pl)EctC_E57A_rev	TTTATACCAAATCAGGGTTTC
pLC56	Tyr-84/Ala	(Pl)EctC_Y84A_for	GGGTATGATG**GC**TGCACTGGATGG
	TAT → GCT	(Pl)EctC_Y84A_rev	GGTGTAATCGGATAGGTTTC
pLC57	His-92/Ala	(Pl)EctC_H92A_for	TCATGAAAAA**GC**TTATCTGCGTGCACG
	CAT → GCT	(Pl)EctC_H92A_rev	CCATCCAGTGCATACATC
pLC58	Cys-104/Ala	(Pl)EctC_C104A_for	GCGTATGGTT**GC**TGTTTTTAATCCGCCTCTGAC
	TGT → GCT	(Pl)EctC_C104A_rev	ATCTGGCTACGTGCACGC
pLC59	Cys-104/Ser	(Pl)EctC_C104S_for	CGTATGGTTT**C**TGTTTTTAATCCGCC
	TGT → TCT	(Pl)EctC_C104S_rev	CATCTGGCTACGTGCACG
pLC67	Trp-21/Ala	(Pl)EctC_W21A_for	TACCACCACC**GC**GAATAGCCGTC
	TGG → GCG	(Pl)EctC_W21A_rev	TCAATATCATCTTTGGTATCC

[a]Mutations introduced into the *P. lautus ectC* gene are indicated with bold letters.

References

1 Delano, W. L. *The PyMol molecular graphics system*. (Delano Scientific, 2002).

2 Afonine, P. V. *et al.* Towards automated crystallographic structure refinement with phenix.refine. *Acta Crystallogr D Biol Crystallogr* **68**, 352-367, doi:10.1107/S0907444912001308 (2012).

3 Widderich, N. *et al.* Biochemistry and crystal structure of the ectoine synthase: a metal-containing member of the cupin superfamily. *PLoS One* **11**, e0151285 (2016).

4 Katoh, K., Rozewicki, J. & Yamada, K. D. MAFFT online service: multiple sequence alignment, interactive sequence choice and visualization. *Brief Bioinform*, 1-7, doi:10.1093/bib/bbx108 (2017).

5 Letunic, I. & Bork, P. Interactive tree of life (iTOL) v3: an online tool for the display and annotation of phylogenetic and other trees. *Nucleic Acids Res* **44**, W242-W245, doi:10.1093/nar/gkw290 (2016).

6 Dunwell, J. M., Culham, A., Carter, C. E., Sosa-Aguirre, C. R. & Goodenough, P. W. Evolution of functional diversity in the cupin superfamily. *Trends Biochem Sci* **26**, 740-746 (2001).

7 Dunwell, J. M., Purvis, A. & Khuri, S. Cupins: the most functionally diverse protein superfamily? *Phytochemistry* **65**, 7-17 (2004).

8 Czech, L. *et al.* Role of the extremolytes ectoine and hydroxyectoine as stress protectants and nutrients: genetics, phylogenomics, biochemistry, and structural analysis. *Genes (Basel)* **9**, 177, doi:10.3390/genes9040177 (2018).
